# Real-Time Optical Mapping of Contracting Cardiac Tissues With GPU-Accelerated Numerical Motion Tracking

**DOI:** 10.3389/fcvm.2022.787627

**Published:** 2022-05-24

**Authors:** Jan Lebert, Namita Ravi, George Kensah, Jan Christoph

**Affiliations:** ^1^Cardiovascular Research Institute, University of California, San Francisco, San Francisco, CA, United States; ^2^German Center for Cardiovascular Research (DZHK e.V.), Göttingen, Germany; ^3^Yale School of Medicine, Yale University, New Haven, CT, United States; ^4^Department for Cardiothoracic and Vascular Surgery, University Medical Center Göttingen, Göttingen, Germany

**Keywords:** computer vision, GPU, optical mapping, cardiac electrophysiology, motion tracking, high-throughput, cell culture

## Abstract

Optical mapping of action potentials or calcium transients in contracting cardiac tissues are challenging because of the severe sensitivity of the measurements to motion. The measurements rely on the accurate numerical tracking and analysis of fluorescence changes emitted by the tissue as it moves, and inaccurate or no tracking can produce motion artifacts and lead to imprecise measurements that can prohibit the analysis of the data. Recently, it was demonstrated that numerical motion-tracking and -stabilization can effectively inhibit motion artifacts, allowing highly detailed simultaneous measurements of electrophysiological phenomena and tissue mechanics. However, the field of electromechanical optical mapping is still young and under development. To date, the technique is only used by a few laboratories, the processing of the video data is time-consuming and performed offline post-acquisition as it is associated with a considerable demand for computing power. In addition, a systematic review of numerical motion tracking algorithms applicable to optical mapping data is lacking. To address these issues, we evaluated 5 open-source numerical motion-tracking algorithms implemented on a graphics processing unit (GPU) and compared their performance when tracking and compensating motion and measuring optical traces in voltage- or calcium-sensitive optical mapping videos of contracting cardiac tissues. Using GPU-accelerated numerical motion tracking, the processing times necessary to analyze optical mapping videos become substantially reduced. We demonstrate that it is possible to track and stabilize motion and create motion-compensated optical maps in real-time with low-resolution (128 x 128 pixels) and high resolution (800 x 800 pixels) optical mapping videos acquired at 500 and 40 fps, respectively. We evaluated the tracking accuracies and motion-stabilization capabilities of the GPU-based algorithms on synthetic optical mapping videos, determined their sensitivity to fluorescence signals and noise, and demonstrate the efficacy of the Farnebäck algorithm with recordings of contracting human cardiac cell cultures and beating hearts from 3 different species (mouse, rabbit, pig) imaged with 4 different high-speed cameras. GPU-accelerated processing provides a substantial increase in processing speed, which could open the path for more widespread use of numerical motion tracking and stabilization algorithms during routine optical mapping studies.

## 1. Introduction

Voltage- or calcium-sensitive optical mapping can resolve electrophysiological wave phenomena in cardiac tissue at high spatial and temporal resolutions (1–6), and can be performed across various spatial scales: in hearts as small as a zebrafish's heart (7–9), in single cardiomyocytes and cell cultures (10–13), and large hearts including human hearts (14). Optical mapping supersedes other imaging techniques in terms of spatial resolution and provides the advantage of non-contact measurement.

However, to date, there are two major technical challenges associated with optical mapping which have so far inhibited exploiting the technique's full potential: First, due to its severe sensitivity to motion, optical mapping is yet widely performed in contraction-inhibited tissues to avoid so-called motion artifacts using pharmacological excitation-contraction uncoupling agents. Motion artifacts arise quickly during optical mapping even with the slightest motion if the measurements are performed in an ordinary fashion: extracting and analyzing the optical signals pixel by pixel without numerical motion tracking (15, 16). As a result, over the past 30 years, the motion has been avoided at all costs in optical mapping studies (17). To overcome this limitation, optical mapping has more recently been combined with numerical motion tracking and stabilization techniques to track and measure fluorescence on the deforming tissue surface in a co-moving reference frame, which effectively inhibits motion artifacts (6, 16, 18–20). Second, the high data rates associated with the fast acquisition rates and high spatial resolutions provided by modern high-speed CCD or CMOS cameras lead to large amounts of data, and the handling, processing, and analysis of the data pose a significant challenge, particularly when numerical motion tracking and stabilization is performed. At present, optical mapping is typically performed at imaging speeds of about 200 to 2, 000 frames per second (fps) with video image sizes of 100 × 100, 128 × 128, or 256 × 256 pixels, and in some applications even with video image sizes greater than 1, 000 × 1, 000 pixels. Accordingly, a single recording results in tens of thousands to millions of parallel optical measurements with data rates ranging in the order of 10–100 million samples per second. With advances in camera sensor technology, the adoption of low-cost high-resolution CMOS cameras (21, 22), and multi-camera imaging systems (23, 24), the data rates will continue to increase, generating large amounts of imaging data that need to be processed.

Myocardial tissue is an electrically excitable tissue, which contracts and deforms in response to electrical excitation, and it is, therefore, crucial to develop measurement tools, with which it is possible to observe electrical wave phenomena and tissue mechanics simultaneously at high spatial and temporal resolutions. Optical mapping combined with computer vision is ideally suited to perform these measurements. The adaptation of computer vision techniques, such as numerical motion tracking, will expand the list of possible applications for optical mapping and provide novel opportunities in cardiovascular research. However, this development also poses a significant challenge in terms of data processing and data handling, which needs to be addressed before the techniques can find more widespread applications.

In this study, we evaluated 5 numerical motion tracking algorithms, which are implemented on a graphics processing unit (GPU), and we applied these algorithms to numerically inhibit motion artifacts in optical mapping recordings. GPU-based algorithms can provide substantial accelerations in terms of processing speed. We evaluated the performance and applicability of these algorithms to various optical mapping data and explored whether their use would allow the numerical tracking and compensation of motion artifacts in real-time. All tested algorithms are open-source and freely available as they are implemented in the open-source computer vision library OpenCV (25). As OpenCV is actively maintained, supported, and available on all major operating systems, it should be possible for other optical mapping practitioners to test and replicate our results or use our findings as guidance for their data analysis.

### 1.1. Tracking Motion in Fluorescence Imaging Videos

**Figure 1:**
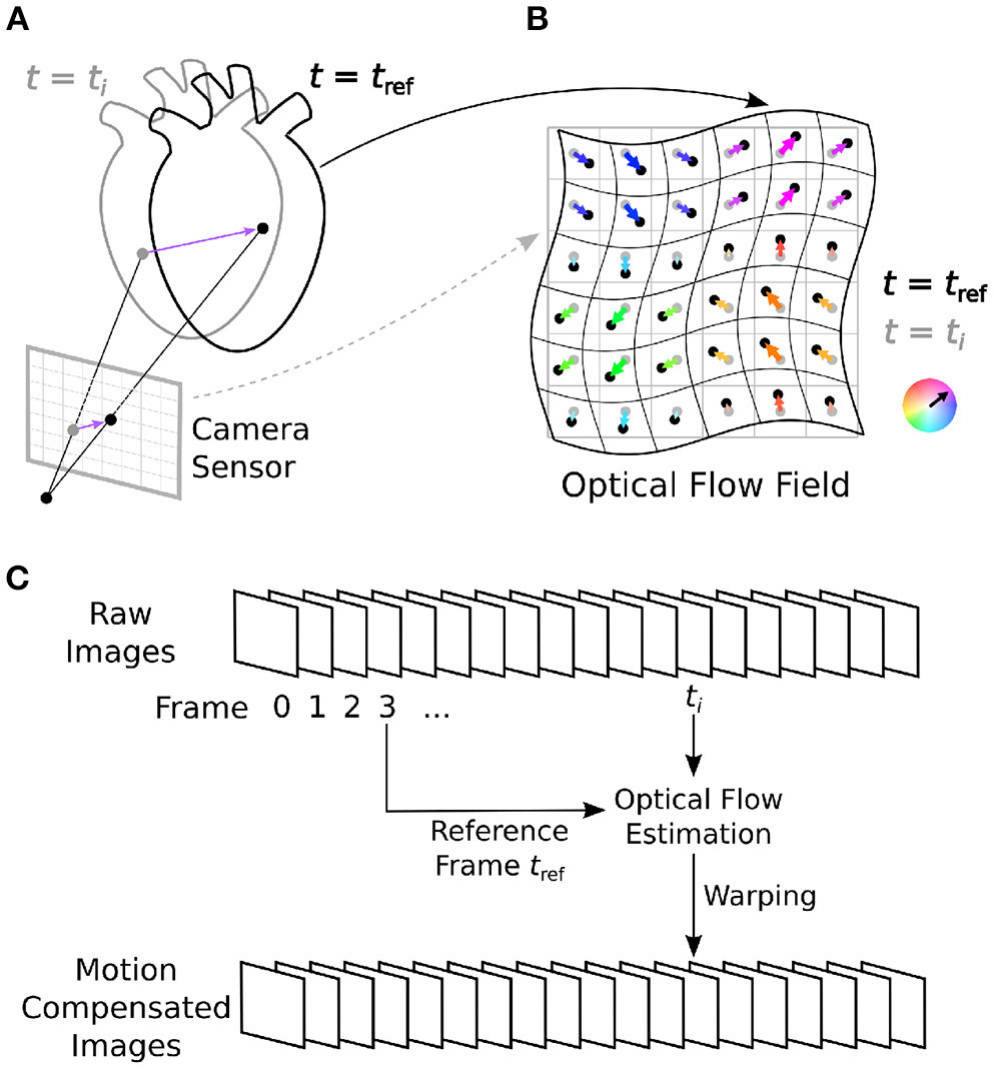
Optical mapping combined with numerical motion tracking for measurement of an action potential or calcium waves in moving and deforming cardiac tissues, such as an isolated heart during voltage-sensitive optical mapping. **(A)** Two-dimensional in-plane displacements on the camera sensor depict the motion of the tissue between two video frames from time *t*_*i*_ to time *t*_*ref*_. **(B)** Optical flow field describing the deformation of the imaged tissue pixel-by-pixel. In **Figure 10** and Supplementary Figures S1, S2, the displacement vector fields are HSV color-coded: the orientation and magnitude of the displacements are depicted by hue (color) and saturation, respectively. **(C)** Numerical motion tracking in a sequence of video images with a calculation of the displacements with respect to a reference frame (first frame or any arbitrary frame).

Tracking motion in optical mapping videos is different from tracking motion in most other applications. The particular properties of optical mapping videos can adversely affect the motion tracking process and add significant error to the tracking results (16). Motion tracking algorithms can be broadly distinguished into two categories: either optical flow or feature tracking techniques. Optical flow algorithms are “dense” motion tracking algorithms in that they track motion in every pixel. Feature tracking algorithms track individual points of interest (landmarks, features, etc.) identifiable in an image. Due to the higher resolution of optical flow algorithms, they are better suited to stabilize motion and inhibit motion artifacts in optical mapping videos, because even the slightest sub-pixel shifts can cause severe motion artifacts. The algorithms we tested are all dense optical flow estimation algorithms, which track optical flow automatically pixel-by-pixel in a two-dimensional scene. For a survey of various numerical motion tracking techniques refer to (26–28). The term optical flow, or image motion, is used in computer vision to refer to the apparent motion or deformation between two images. This two-dimensional image motion is the projection of the three-dimensional motion or deformation of objects relative to a camera sensor onto its image plane, refer to Figure 1. An optical flow field is a two-dimensional vector field describing the displacements of brightness patterns from one image to another. Optical flow can be caused relative motion as well as the deformation of objects. Relative motion is either movement of objects with respect to a static viewpoint or movement of the viewpoint or both.

One key assumption commonly made by optical flow algorithms is brightness constancy, which means that the intensity of objects in a scene does not change over time (29, 30). With optical mapping, however, this assumption is violated because the fluorescent signals, which are caused by (voltage- or calcium-sensitive) fluorescent markers, can change significantly over time. Consequently, the visual appearance of fluorescing tissue can change from video frame to video frame, which makes determining the optical flow and tracking motion between frames challenging. While brightness constancy is almost never perfectly fulfilled in most other real-life scenarios as well, during optical mapping the brightness changes can become particularly severe as they are an intended feature and central part of the measurement: the stronger the brightness changes, the better the measured signal. In previous studies, refer to review by Nesmith et al. (31), the numerical tracking was either facilitated by attaching physical markers to the tissue (18, 32, 33) or performed entirely without markers solely tracking the tissue's natural features (6, 15, 16, 19, 20, 34–36). Particularly in the latter case, when solely the fluorescing tissue is imaged, brightness changes can lead to tracking artifacts (16), and it is, therefore, critical to carefully consider these brightness changes when assessing the performance of numerical motion tracking algorithms. In this study, we tested the GPU algorithm's sensitivity to noise and fluorescence-induced brightness changes.

## 2. Methods

We evaluated 5 dense optical flow algorithms, refer to Section 2.2, which are part of the popular, freely available, open-source computer vision library OpenCV (25): 1) Lucas and Kanade (37) with OpenCV modifications, 2) Farnebäck (38), 3) Brox (39), 4) TV-L1 (40), and 5) the ‘NVIDIA Optical Flow SDK'. All algorithms are executed on a GPU. We applied the algorithms to track and stabilize motion and deformation in voltage- and calcium-sensitive optical mapping videos recorded with 4 different high-speed cameras, as well as in synthetic optical mapping videos which we generated in computer simulations. In particular, the synthetic data provides ground-truth displacement data, which allows a precise evaluation of the results of the tracking algorithms. We evaluated and compared the performance of the algorithms with regard to processing speed and accuracy on video data with different noise levels and fluorescence intensities. We also compared the processing speeds of two GPU-based algorithms with their corresponding CPU implementations. Processing was performed on a workstation with Ryzen Threadripper 3970X 3.7GHz CPU (AMD Inc., USA) equipped with 32 cores and a Geforce RTX 3070 GPU (NVIDIA Corp. USA). The backbone of numerical motion tracking methods is dense optical flow motion tracking algorithms, which estimate motion or displacements in every pixel of an image, from one image to another, in a fully automatic fashion. All numerical routines were implemented in Python and/or C++.

### 2.1. Imaging Data

#### 2.1.1. Synthetic Data

To evaluate the tracking accuracy of the motion tracking algorithms, we generated synthetic optical mapping videos as described in Christoph and Luther (16). In short, the synthetic imaging data was created by deforming grayscale video still frames showing a heart during an optical mapping experiment using an electromechanical computer simulation. The video frame was not only deformed but the simulated action potential wave patterns were superimposed as intensity decreases over the parts of the images showing the heart tissue, refer to **Figure 10A**). Furthermore, the images were deformed as if the tissue was deforming in response to the action potential wave patterns. The deformed images were subsequently resampled. Because the deformations are simulated, the actual displacements are known in every pixel as ground-truth displacement data. Accordingly, we created a dataset consisting of synthetic image pairs showing the tissue in one and another deformed state at times *t*_1_ and *t*_2_ and the ground-truth deformation vector field between the two images. We denote the electrical excitation of the simulation at time *t* as *v*(*x, y, t*) and the deformation with respect to the initial mechanical configuration as $\vec{d}\left(x,y,t\right)$. A synthetic image Ĩ(*x, y, t*) for a given fluorescence strength *f*, which denotes the fractional change in fluorescence Δ*F*/*F* exhibited by fluorescent probes in optical mapping experiments, is generated as follows:


(1) $$\tilde{I}(x,y,t)=(1+f\cdot v(x,y,t))\cdot I_{\text{texture}}(x,y)$$

**Figure 2:**
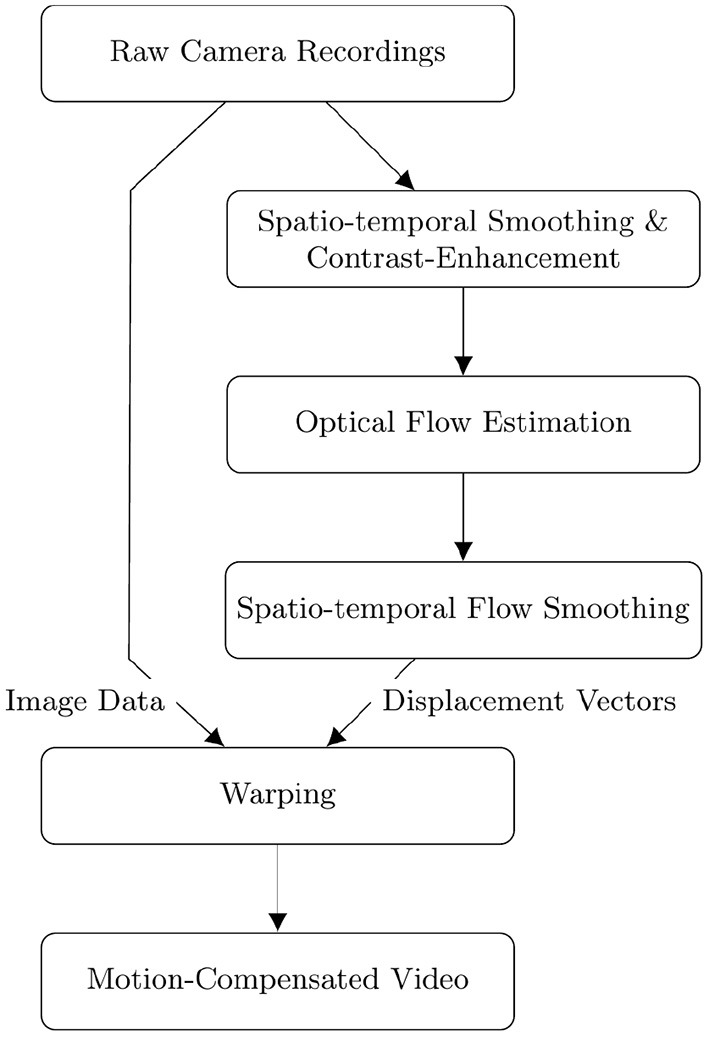
Flow diagram depicting the sequential order of the fully automatic numerical pre- and post-processing and tracking of optical mapping data. Raw videos are smoothed simultaneously in space and time and then contrast-enhanced [also refer to Christoph and Luther (16)] before performing the optical flow estimation. The displacement vectors resulting from the optical flow estimation are smoothed in space and time before being used to warp the original raw video frames to obtain a motion-compensated video.

To generate an image pair, we choose the experimental image *I*_texture_(*x, y*), onto which the action potential wave patterns are superimposed, and two simulation time points *t*_1_ and *t*_2_ at random. The first image of an evaluation image pair is Ĩ(*x, y, t*_1_), the second image is the image Ĩ(*x, y, t*_2_) deformed by the displacement vectors $\vec{u}\left(x,y\right)=\vec{d}\left(x,y,t_{2}\right)-\vec{d}\left(x,y,t_{1}\right)$. Here, the displacement vector field $\vec{u}\left(x,y\right)$ is used to warp one image into the other. The central step in the motion-compensation algorithm is the estimation of the deformation vector field $\vec{u}\left(x,y\right)$ between the first and second image using an optical flow algorithm, refer to Figure 2.

#### 2.1.2. Experimental Data

The experimental imaging data processed and analyzed in this study consists of voltage-sensitive optical mapping videos of a mouse and a rabbit heart imaged *ex vivo* with a Basler acA720-520um camera (720 × 540 pixels full resolution), voltage-sensitive optical mapping videos of a rabbit heart imaged *ex vivo* with a Brainvision Scimedia MiCAM N256 camera (256 × 256 pixels full resolution) and an IDS μEye UI-3060CP-M-GL camera (1, 936 × 1, 216 pixels full resolution), voltage-sensitive optical mapping videos of a pig heart imaged *ex vivo* with a Photometrics Teledyne Evolve 128 camera (128 × 128 pixels full resolution), and calcium- and voltage-sensitive optical mapping videos of a cardiomyocyte culture, differentiated from human induced pluripotent stem cells (iPSCs), imaged *in vitro* with an IDS μEye UI-3060CP-M-GL camera (1, 936 × 1, 216 pixels full resolution). In addition, we discuss calcium-sensitive optical mapping data of an isolated stomach strip imaged *in vitro* with an Oxford Instruments Andor camera (1, 280 × 1, 080 pixels full resolution) which we analyzed in another study with our methodology (36). All tissues contract and deform strongly as they were imaged in the absence of pharmacological excitation-contraction uncoupling agents.

### 2.2. Optical Flow Estimation Motion Tracking Algorithms

We tested 5 different GPU-based dense optical flow estimation algorithms implemented in the popular open-source OpenCV computer vision library (25), version 4.4.0. The estimation of optical flow between two images is an ill-posed inverse problem, and a solution, a vector field describing the affine transformation of one image into another, can be approximated with optimization techniques. To find the appropriate flow field between two images, optical flow algorithms employ a variety of assumptions on both the data, such as the brightness constancy assumption, as well as the solution, such as smoothness constraints. The choice of assumptions and optimization techniques is an important degree of freedom in designing optical flow algorithms and has led to a wide range of methods over the past several decades (26).

Lucas and Kanade (37) and Horn and Schunck (41) are seminal works in the development of optical flow methods, which broadly represent two different categories of motion tracking algorithms upon which most modern algorithms are based. Lucas and Kanade (37) is a so-called *local method*: it computes the flow vectors for all pixels separately. It assumes that all pixel intensity changes between the two images are caused by the movement of the underlying objects and that the flow is constant within a local neighborhood of each pixel. Horn and Schunck (41) is a variational method for optical flow estimation: it is a *global method* that estimates the optical flow for all pixels jointly. It computes the optical flow as the solution to a minimization problem using regularization, relying on the assumption that the brightness of a pixel is constant over time.

- Lucas-Kanade: We used the ‘DensePyrLKOpticalFlow' implementation in OpenCV, which extends the Lucas and Kanade (37) algorithm to give dense optical flow estimates, using an iterative coarse-to-fine scheme (42). This coarse-to-fine approach is a commonly used concept in optical flow estimation to address large displacements: by repeatedly reducing the resolution of the images over several steps, and starting from the smallest image resolution. The Lucas-Kanade algorithm is applied at each resolution and then successively refines the estimated optical flow by applying the Lucas-Kanade algorithm to the higher resolution images.
- Farnebäck: The algorithm introduced in Farnebäck (38) is a local method similar to Lucas-Kanade. Among other differences, it assumes an affine flow, as opposed to a constant flow, in the local pixel neighborhood.
- Brox: Brox et al. (39) proposed a variational method for optical flow estimation, which aims to allow larger displacements compared to Horn and Schunck (41).
- TV-L1: There exist several variational algorithms which are based on total variation (TV) regularization using the *L*_1_ norm to preserve discontinuities in the optical flow field. Here, we use the algorithm introduced in Zach et al. (40), whose implementation is described in Sánchez Párez et al. (43).
- NVIDIA: In 2019, the NVIDIA Corporation introduced the ‘NVIDIA Optical Flow SDK'^1^, a software library for the estimation of the relative motion of pixels between images. The library relies on dedicated hardware capabilities of NVIDIA Turing and Ampere GPUs for the optical flow computation. The library requires a minimum image size of approximately 160 × 160 pixels, we scale images sized 128 × 128 pixels to 256 × 256 pixels to overcome this limitation. NVIDIA has not detailed how the optical flow algorithm works but provides the source code upon request.^2^

With all optical flow algorithms, we used the default parameters in OpenCV. The OpenCV implementations of all algorithms except the Brox algorithm require 8-bit images as input. The Brox algorithm also supports 16-bit images. Optical mapping recordings are usually captured using 12-bit (pixel intensity values between 0 and 4,095), 14-bit (pixel intensity values between 0 and 16,383), or 16-bit (pixel intensity values between 0 and 65,535) camera sensors. The synthetic camera images we generated are 16-bit images. With the exception of the Brox optical flow algorithm, we reduced the generated images to the 8-bit range (values between 0 and 255) for optical flow estimation. However, there is no loss in image precision for the motion-compensated (warped) videos as the resulting 32-bit floating-point tracking data is then applied to the original data.

### 2.3. Tracking Accuracy

The tracking accuracy or tracking error of the algorithms was determined by calculating the average end-to-end point error (EPE), which is the average Euclidean distance between the displacement vectors estimated by the motion tracking algorithms and the ground-truth or reference displacement vectors being the output of simulations or a reference algorithm, respectively. The EPE is averaged over all pixel locations for every displacement vector and then averaged over all vectors in 20,000 video images correspondingly. In this study, the displacement vector fields are shown as color-coded HSV maps instead of vector fields, the hue (color) indicating the orientation and the saturation showing the magnitude of the displacements, respectively, refer to Figure 1.

### 2.4. Pre- and Post-processing

Accurate motion tracking and motion artifact compensation of optical mapping data typically require pre-processing of the imaging data and post-processing of computed displacement vector fields. Figure 2 depicts a flow diagram of the computational steps for computing a motion-compensated video of a raw optical mapping video. The diagram follows the procedure detailed in Christoph and Luther (16). In short, the pre-processing of experimental data consists of i) smoothing the video data in both space and time (for further details refer to results section) and ii) computing a locally contrast-enhanced version of the original video. The contrast-enhanced video *I*_*c*_(*x, y, t*) is obtained by renormalizing each pixel's intensity value *I*(*x, y, t*) using the maximal and minimal pixel intensities within a small circular region *S*(*x, y, t*) around the pixel:


(2) $$\begin{array}{r} I_{c}\left(x,y,t\right)=\frac{I\left(x,y,t\right)-\text{min}\left(S\left(x,y,t\right)\right)}{\text{max}\left(S\left(x,y,t\right)\right)-\text{min}\left(S\left(x,y,t\right)\right)} \end{array}$$

As a result, local grayvalue patterns representing the tissue become amplified yielding videos with maximal spatial contrast, refer to **Figure 13**. At the same time, time-varying signals caused by fluorescent reporters are inhibited in contrast-enhanced videos, refer to Christoph and Luther (16). This can reduce tracking artifacts caused by the violation of the brightness constancy assumption in the optical flow estimation, refer to **Figure 10**. The necessity of contrast-enhancement and the effect on different optical flow algorithms is analyzed in Section 3.2. The optical flow algorithm then estimates the displacement vector field $\vec{u}\left(x,y,t\right)$ of the contrast-enhanced video with respect to a reference frame as shown in Figure 1. The displacement vector fields $\vec{u}\left(x,y,t\right)$ are then spatio-temporally smoothed (for further details refer to results section) to further reduce tracking artifacts and enforce temporal smoothness. Finally, the motion-compensation video is computed by warping the original raw video with the smoothed displacement vector fields. EPEs were computed on displacement vector fields that were not smoothed.

## 3. Results

**Figure 3:**
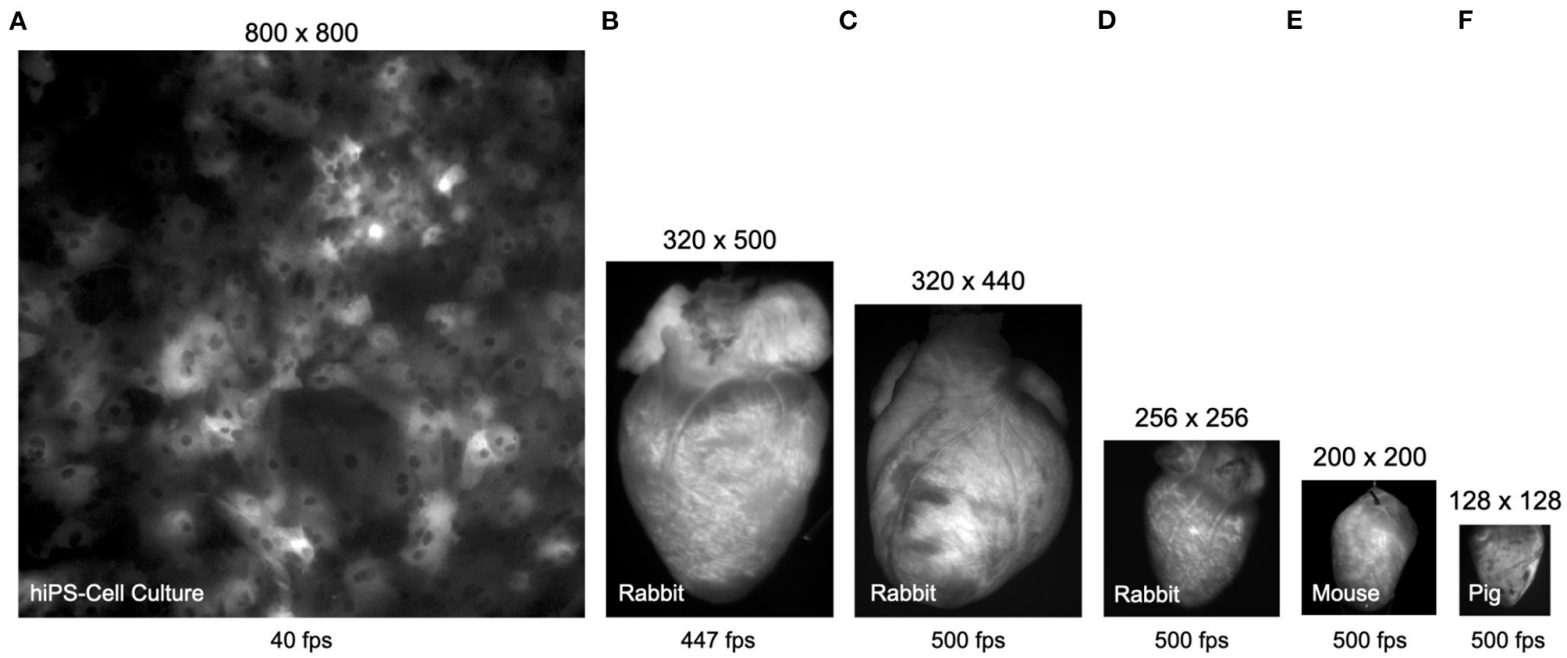
Comparison (to scale) of video data obtained with different cameras in calcium- and voltage-sensitive optical mapping experiments. All hearts were stained with voltage-sensitive fluorescent dye Di-4-ANEPPS. **(A)** Human iPSC-derived cardiomyocyte monolayer expressing the genetically encoded calcium indicator GCaMP-X imaged at 40 fps with 800 × 800 pixel resolution (0.74mm field of view) using an IDS μEye UI-3060CP-M-GL camera (refer to Supplementary Video 6). **(B)** Rabbit heart imaged at 447 fps with 320 × 500 pixel resolution using an IDS μEye UI-3060CP-M-GL camera (refer to Supplementary Video 3). **(C)** Rabbit heart imaged at 500 fps with 320 × 440 pixel resolution using a Basler acA720-520um camera (refer to Supplementary Videos 1, 2). **(D)** Rabbit heart imaged at 500 fps with 256 × 256 pixel resolution using a Brainvision Scimedia MiCAM N256 camera (refer to Supplementary Video 9). **(E)** Mouse heart imaged at 500 fps with 200 × 200 pixel resolution (cropped from original video imaged with 720 × 320 pixels) using a Basler acA720–520 um camera (refer to Supplementary Video 4). **(F)** Porcine heart imaged at 500 fps with 128 × 128 pixel resolution using a Teledyne Photometrics Evolve 128 camera. The video data in **(A,F)** can be processed in real-time, faster than the acquisition speeds at 40 and 500 fps, respectively, using GPU-accelerated processing, refer to **Figures 8**, **9**.

**Figure 4:**
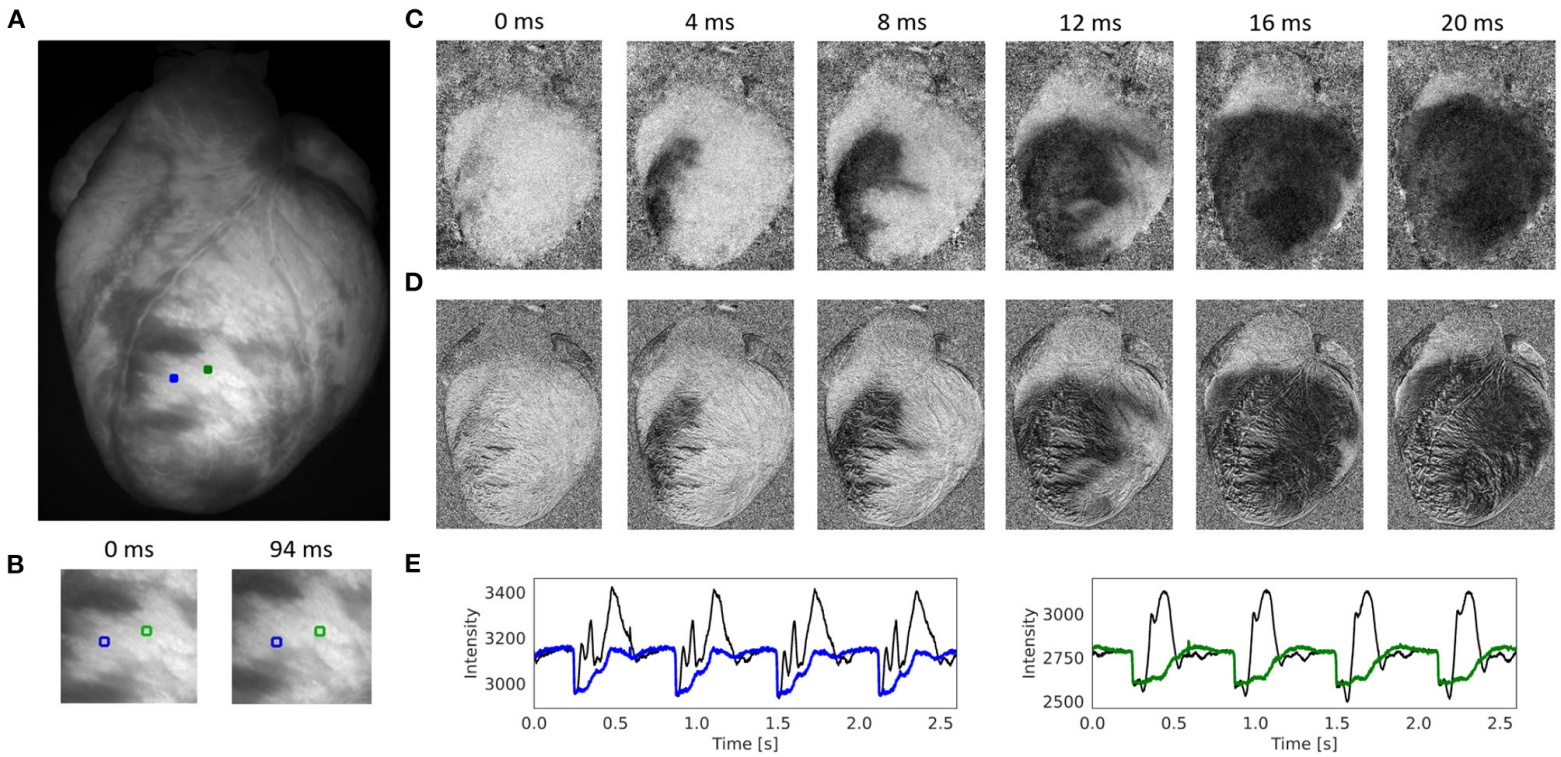
Contracting rabbit heart imaged during sinus rhythm using voltage-sensitive optical mapping (staining with Di-4-ANEPPS), refer to Supplementary Video 1. Motion artifacts were inhibited using GPU-accelerated numerical motion tracking and motion compensation. **(A)** Video image from original recording (500 fps, 320 × 440 pixels, Basler acA720-520um camera). ROI (green and blue, 6 × 6 pixels) used to extract optical traces shown in **(E)**. **(B)** Motion and deformation of heart surface around ROIs shown in **(A)** during diastole and systole (region 100 × 100 pixels), the movement is about 25 pixels (13% of the total image width). **(C)** Series of pixel-wise normalized (20 ms sliding window) optical maps obtained after numerical motion tracking and motion compensation using the GPU-accelerated Farnebäck algorithm. Action potential wave (black: depolarized tissue) propagating from bottom left to top right across the left ventricle. **(D)** Series of pixel-wise normalized (20 ms sliding window) optical maps without numerical motion tracking and motion compensation. Without motion compensation, the optical maps are distorted by motion artifacts. The action potential wave is yet visible due to the short sliding-window normalization and a relatively strong signal (|Δ*F*/*F*|≈7%). **(E)** Optical traces averaged from ROIs in **(A)** before (black) and after (blue and green) numerical motion compensation using Farnebäck GPU-based motion tracking algorithm. Motion artifacts become significantly reduced after numerical tracking and stabilization. However, residual illumination-related motion artifacts cannot be overcome with numerical motion tracking alone, refer to Kappadan et al. (20).

**Figure 5:**
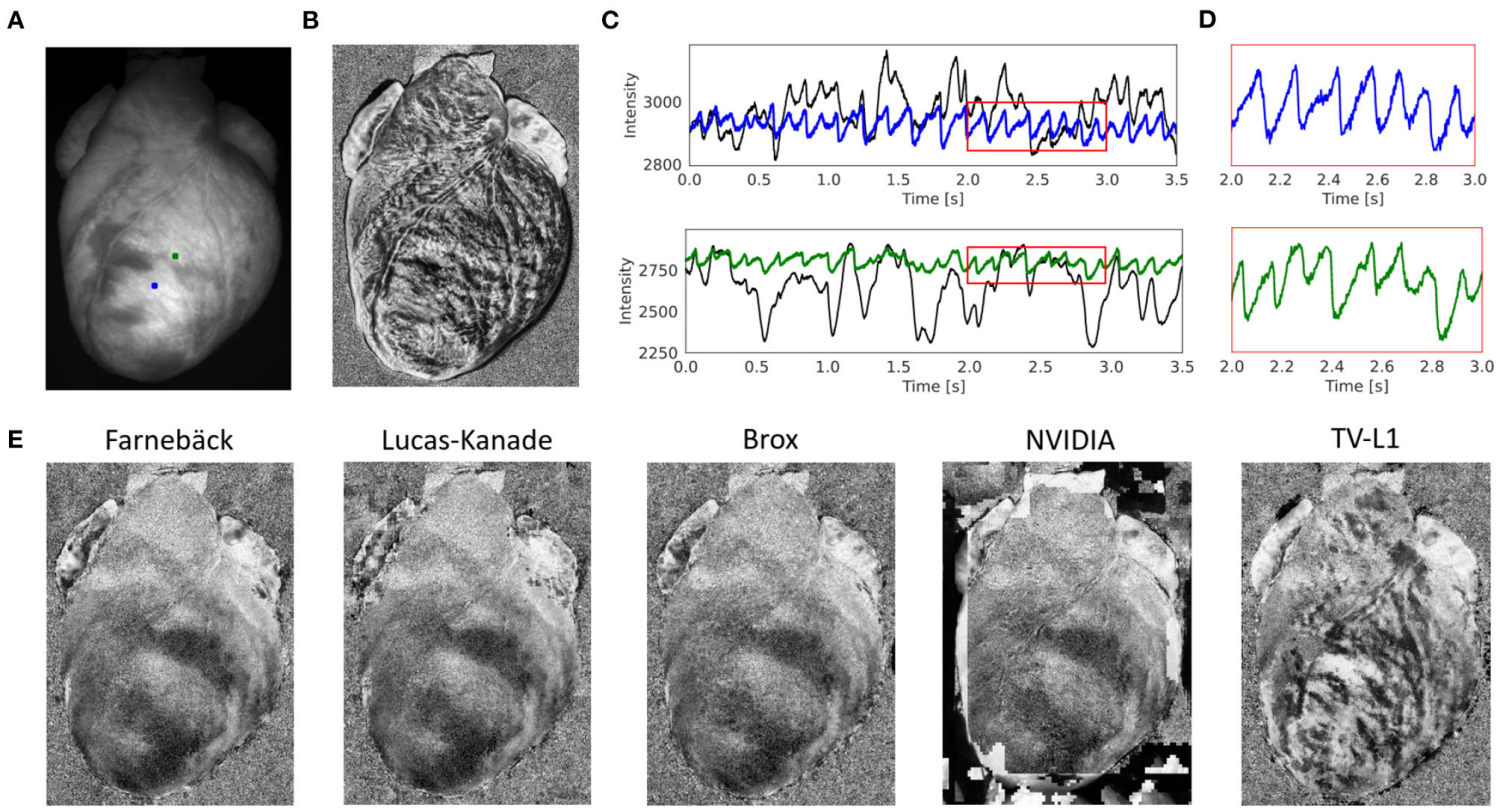
Contracting rabbit heart imaged during ventricular fibrillation (VF) using voltage-sensitive optical mapping (staining with Di-4-ANEPPS). Motion artifacts were inhibited using GPU-accelerated numerical motion tracking and motion compensation. **(A)** Video image from original recording (500 fps, 320 × 440 pixels, Basler acA720–520 um camera). ROI (green and blue, 6 × 6 pixels) used to extract optical traces shown in **(C)**. **(B)** Pixel-wise normalized (0.5 s sliding window) optical map without numerical motion tracking and motion compensation. Without motion compensation, the optical maps are dominated by motion artifacts. Action potential waves are not visible. **(C)** Optical traces measured from two sites shown in **(A)** before (black) and after (blue and green) numerical motion compensation using Farnebäck GPU-based motion tracking algorithm. Motion artifacts become significantly reduced. Traces averaged over the two rectangular regions shown in **(A)**. **(D)** Close-up of motion-corrected optical traces in **(C)** showing a series of action potentials (downstrokes correspond to action potential upstrokes due to staining). During VF, residual illumination-related motion artifacts are small due to the minimal motion of the heart, refer to Kappadan et al. (20). **(E)** Motion-compensated, pixel-wise normalized optical maps derived with 5 different motion tracking algorithms (Farnebäck, Lucas-Kanade, Brox, NVIDIA, TV-L1; all GPU, sorted from left to right by robustness/accuracy) show vortex-like action potential waves (dark) on the fibrillating ventricular surface, refer to Supplementary Video 2.

**Figure 6:**
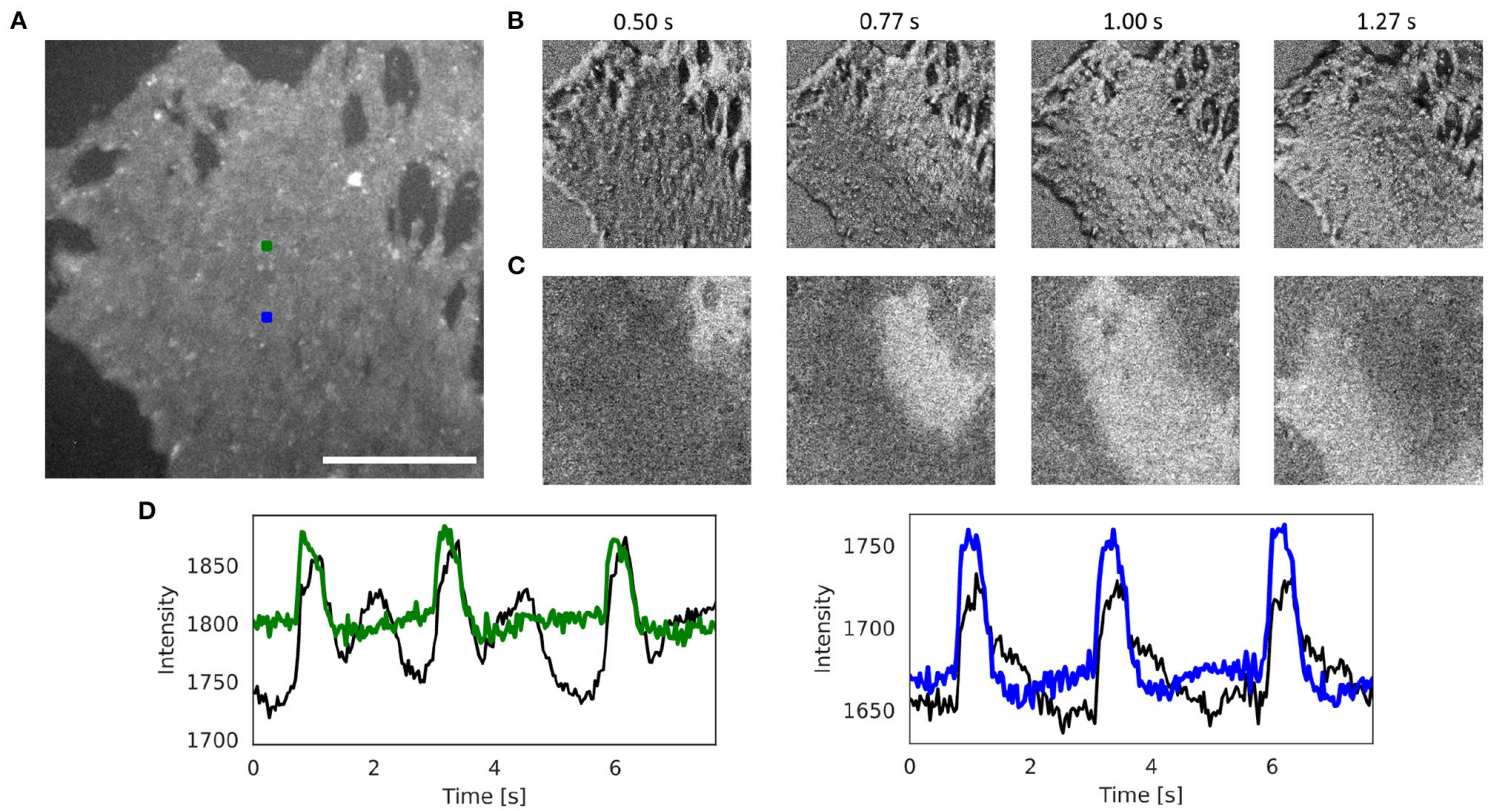
Numerical motion tracking and motion artifact compensation performed with Farnebäck GPU-based motion tracking algorithm with optical mapping video of strongly contracting and deforming human iPSC-derived cardiomyocyte culture stained with voltage sensitive dye (FluoVolt), refer to Supplementary Videos 5, 8. **(A)** Cropped video image (400 × 400 pixels, scalebar 1mm) from original recording (40fps, 800 × 800 pixels, IDS μEye UI-3060CP-M-GL camera). ROI (green and blue, 10 × 10 pixels) used to extract optical traces shown in **(D)**. **(B)** Pixel-wise normalized (with 4 s sliding window) video without motion compensation with motion artifacts. **(C)** Pixel-wise normalized video after motion compensation without motion artifacts. The action potential wave is visible as a bright wave. **(D)** Optical traces averaged from ROIs in **(A)** obtained from videos before (black) and after (red and blue) motion tracking and artifact compensation (y-axis: intensity counts).

**Figure 7:**
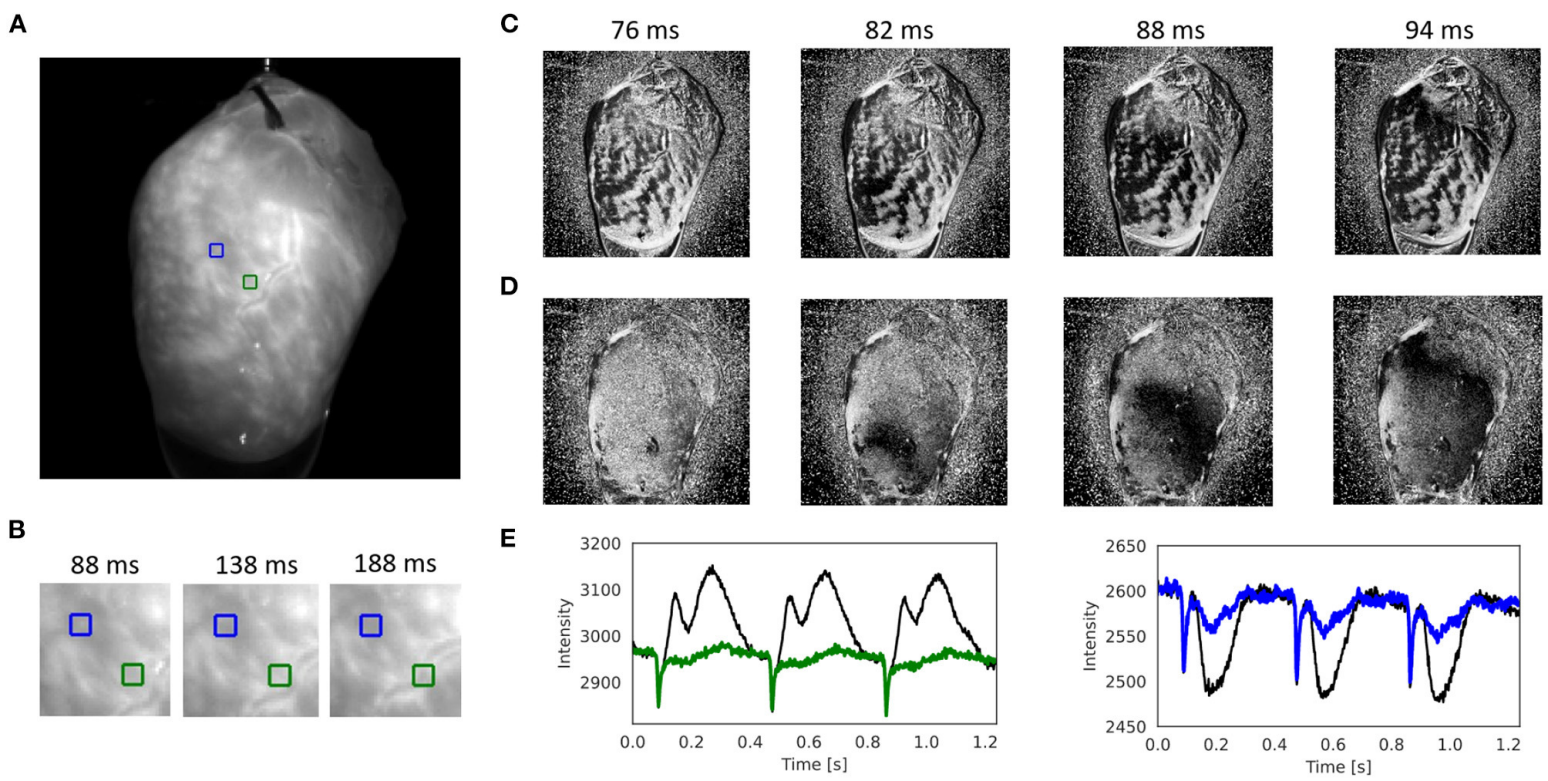
Numerical motion tracking and motion artifact compensation performed with Farnebäck GPU-based motion tracking algorithm with optical mapping video of mouse heart during sinus rhythm with voltage sensitive-staining (Di-4-ANEPPS), refer to Supplementary Videos 4, 7. **(A)** Cropped video image (200 × 200 pixels) from the original recording (500fps, 720 × 320 pixels, Basler acA720-520um camera). ROI (green and blue, 6 × 6 pixels) used to extract optical traces shown in **(E)**. **(B)** Motion and deformation of heart surface around ROIs shown in **(A)** over time (40 × 40 pixels). **(C)** Pixel-wise normalized video without motion compensation with motion artifacts. **(D)** Pixel-wise normalized video after motion tracking and compensation without motion artifacts. The action potential wave is visible as a dark wave. **(E)** Optical traces averaged from ROIs in **(A)** obtained from videos before (black) and after (green and blue) motion tracking and artifact compensation.

**Figure 8:**
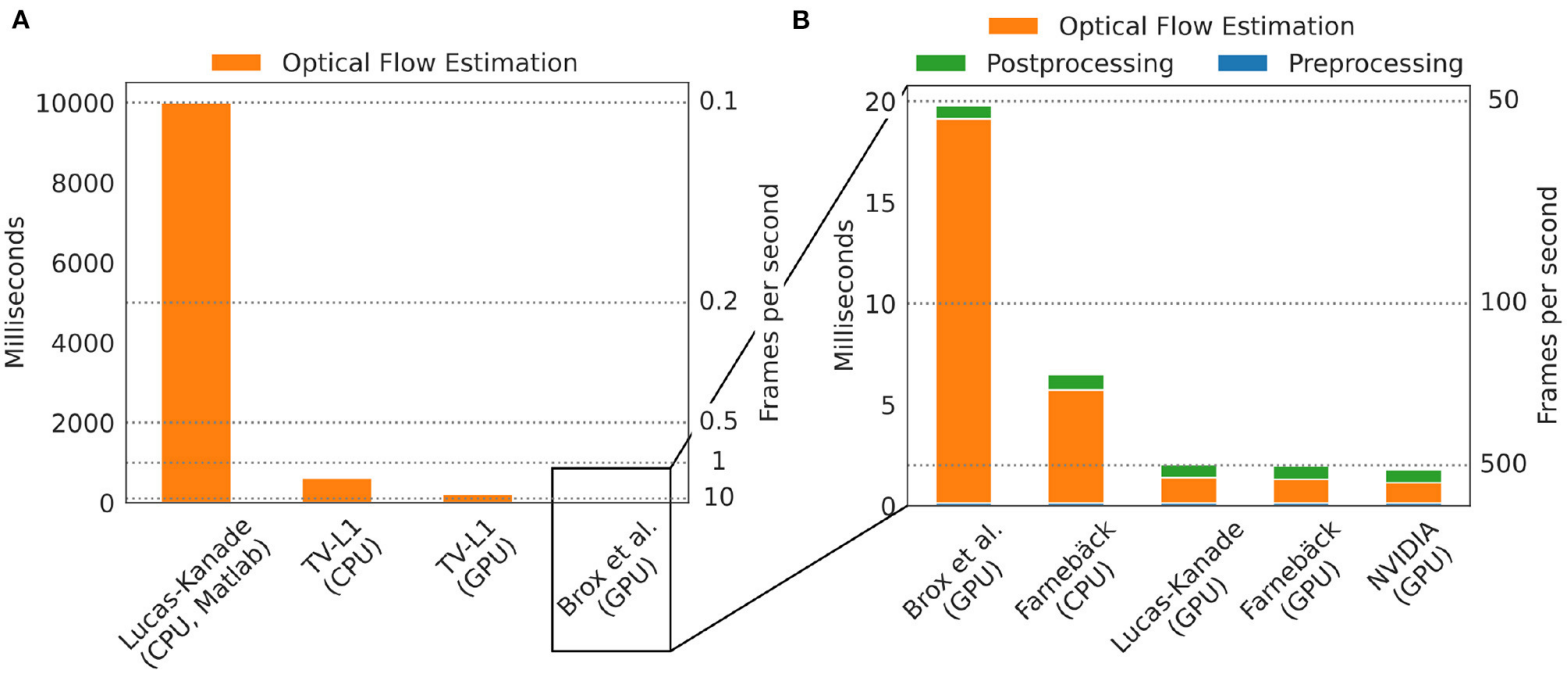
Comparison of processing speeds for tracking motion in optical mapping recordings using different CPU- and GPU-based optical flow estimation algorithms. We compared processing times measured in milliseconds per frame pair for 128 × 128 pixel video images (averaged over 10, 000 video image pairs) including pre- and post-processing to obtain motion-stabilized videos. Processing was performed on a workstation with AMD Ryzen Threadripper 3970X 3.7GHz CPU and an NVIDIA Geforce RTX 3070 GPU. **(A)** Comparison of a single-core CPU-based Lucas-Kanade reference algorithm (used in Christoph et al. (6) and Christoph and Luther (16)) implemented in Matlab (0.1 fps) with TV-L1 (1.6 fps on CPU, 4.6 fps on GPU, both implemented in Python with OpenCV). **(B)** The remaining algorithms provide even higher processing speeds with up to a 5, 000-fold increase in processing speed: Brox 51 fps (53 fps), Lucas-Kanade 489 fps (795 fps), Farnebäck 148 fps (176 fps) on CPU and 510 fps (852 fps) on GPU, NVIDIA SDK 560 fps (1,000 fps), stated as effective frames per second (fps) with (and without) pre- and post-processing. Preprocessing: contrast-enhancement and spatiotemporal data smoothing as described in Christoph and Luther (16). Post-processing: spatiotemporal smoothing of the computed flow fields and using the flow fields to obtain motion-compensated images (warping). The pre- (0.14 ms per framepair) and post-prossing (0.65 ms per framepair when using GPU-based warping, 0.88 ms when using CPU-based warping) time is the same for all algorithms and is performed on the CPU with parallelization.

**Figure 9:**
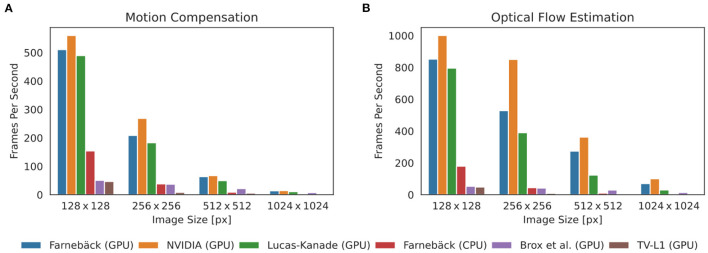
Comparison of processing speeds achieved with 5 different motion tracking algorithms (5 GPU + 1 CPU version): Farnebäck (GPU), NVIDIA SDK (GPU), Lucas-Kanade (GPU), Farnebäck (CPU), Brox (GPU), and TV-L1 (GPU). Processing speeds for video images with 128 × 128, 256 × 256, 512 × 512, and 1024 × 1024 pixels, respectively. Results were obtained with an NVIDIA Gefore RTX 3070 GPU (approx. $500). **(A)** Processing speeds (frames per second) with pre- and post-processing (contrast-enhancement, spatio-temporal smoothing, motion tracking, spatio-temporal smoothing of displacement vector fields, and warping of videos) to obtain motion-stabilized videos. Motion-stabilized videos can be produced in real-timeat 500fps with the Farnebäck, NVIDIA, and Lucas-Kanade GPU algorithms with small videos with 128 × 128 pixels. **(B)** Processing speeds (frames per second) without pre- and post-processing tracking only motion. The Farnebäck CPU algorithm (running on 1 core) is competitive with the GPU-based algorithms with small video images (approximately 3x slower).

Figure 3 shows typical examples of optical mapping data acquired with 4 different high-speed cameras, the μEye UI-3060CP-M-GL camera by IDS, the acA720-520um camera by Basler, the Micam N256 camera by Brainvision Scimedia, and the Evolve 128 camera by Teledyne Photometrics. The 4 cameras, among others, are commonly used in optical mapping studies in basic cardiovascular research. The images show to scale and illustrate the spatial and temporal resolutions that can be achieved with most modern high-speed cameras used in optical mapping studies (although the Micam N256 camera achieves higher imaging speeds than shown). The images are shown in Figure 3A a human iPSC-derived cardiomyocyte monolayer culture, in Figures 3B–D rabbit hearts, in Figure 3E a mouse heart, and in Figure 3F a pig heart. All hearts were stained with the voltage-sensitive fluorescent dye Di-4-ANEPPS. We processed all videos using the Farnebäck motion tracking algorithm, which we determined to be best suited for processing optical mapping data, refer to Figures 4–**7**, Supplementary Videos 1–9, and Supplementary Material. With the high processing speeds provided by GPU-based motion tracking algorithms, we were able to process the video data from low-cost CMOS cameras (the ones by IDS and Basler), which produce larger video images, at high (near real-time) speeds, refer to Figures 4–**7**. Furthermore, we were able to process the low-resolution video data (128 × 128 pixels, acquired at 500 fps) shown in Figure 3F on the right, as well as the high-resolution video data (800 × 800 pixels, acquired at 40 fps) shown in Figure 3A on the left in real-time (using an NVIDIA RTX 3070 GPU, approximately $500), refer to also Section 3.1 and **Figures 8**, **9** for more details on processing speeds. Overall, the GPU-accelerated processing makes the analysis of high-resolution video data and real-time analysis feasible.

Figure 4 and Supplementary Video 1 show the performance of the Farnebäck GPU algorithm with sinus rhythm data imaged in an isolated, contracting rabbit heart. The optical maps in Figures 4C,D show the effect of the numerical tracking and motion artifact inhibition, while an action potential wave (black: voltage-sensitive staining with Di-4-ANEPPS) propagates across the left ventricle. While motion artifacts are suppressed in Figure 4C due to the motion tracking, Figure 4D shows the same but non-stabilized data that was not tracked. The non-stabilized optical maps contain strong motion artifacts. Note that the optical maps were pixel-wise normalized (with a sliding window length of 10 frames or 20 ms), also refer to Section 2.4. Figure 4E shows the optical traces measured in the two locations shown in Figures 4A,B before (black) and after (blue and green) numerical motion tracking and motion artifact inhibition. The uncorrected optical traces reflect in large parts motion of the tissue (aside from the rapid downstrokes) and correspond almost solely to motion artifacts, also refer to **Figure 7**. The corrected, motion-compensated optical traces exhibit an action potential-like shape. While motion artifacts are significantly reduced in the motion-compensated optical traces, they yet contain residual illumination-related motion artifacts, which cannot be compensated with numerical motion tracking and stabilization alone, also refer to Kappadan et al. (20) and Section 4.1. Illumination-related motion artifacts are suppressed in Figure 4C due to the short sliding window used with the pixel-wise normalization, c.f. **Figure 7** and Supplementary Figure S4 for a comparison with a longer window.

Figure 5 and Supplementary Videos 2, 3 demonstrate the performance of the numerical motion tracking and motion-stabilization procedure on optical mapping data showing a rabbit heart during ventricular fibrillation. During ventricular fibrillation, the contractile and translational motion of the heart is significantly reduced and illumination-related motion artifacts play less of a role than during sinus rhythm. Figure 5A shows the original video image. Figure 5B shows the pixel-wise normalized (0.5 s sliding window) but otherwise unprocessed non-tracked video image. Due to the motion, action potential waves are not visible and the pixel-wise normalized optical maps are dominated by motion artifacts (motion artifacts are also stronger due to the longer sliding window than in Figure 4). Figures 5C,D show the optical traces measured in the two locations in Figure 5A before (black) and after (blue and green) numerical motion tracking and stabilization. Without numerical motion compensation, the fluctuations of the signal are much larger (approximately 3–5x) and more erratic than with motion compensation. By contrast, with motion compensation, the signals are more regular with more clearly defined action potential upstrokes and a more uniform amplitude across action potentials. **Figure 11A** and Supplementary Figure S5A show other examples of optical traces measured during ventricular fibrillation on the contracting heart surface (of a pig heart recorded with the Photometrics camera). Residual baseline drifts in the tracked motion-corrected signals are caused by residual illumination-related motion artifacts and by the relatively long sliding window during the (temporal) pixel-wise normalization. Figure 5E shows motion-corrected optical maps, which we retrieved with the 5 different GPU motion tracking algorithms: Farnebäck, Lucas-Kanade, Brox, NVIDIA, and TV-L1 (from left to right and sorted by robustness/accuracy). We obtained good results with the Farnebäck and the Lucas-Kanade algorithm, also refer to Supplementary Video 2.

Figure 6 and Supplementary Videos 5, 8 show the effectiveness of the numerical motion tracking and motion-stabilization procedure using the Farnebäck motion tracking algorithm with cell culture data. The human iPSC-derived cardiomyocyte culture deforms very strongly. It was stained with the fluorescent dye FluoVolt (resulting in a positive fractional change in fluorescence) and was imaged at 40 fps with 800 × 800 pixel resolution using the IDS camera. Figure 6A shows the original video image. Figure 6B shows the pixel-wise normalized (6 s sliding window) but otherwise unprocessed non-tracked video image. Due to the motion, the pixel-wise normalized optical maps are superimposed by motion artifacts and the action potential wave is barely visible. Figure 6C shows the tracked and motion-stabilized pixel-wise normalized (6 s sliding window) optical maps without motion artifacts. The action potential is clearly visible as it propagates from the top right to the bottom left corner. Supplementary Video 8 demonstrates how the different motion tracking algorithms perform on the data. Figure 6D shows the optical traces measured in the two locations in Figure 6A before (black) and after (blue and green) numerical motion tracking and stabilization.

Figure 7, Supplementary Figure S4, and Supplementary Videos 7, 9 show other examples of sinus rhythm in a contracting mouse and rabbit heart, respectively. As in Figures 4–6, the motion was tracked and the effectiveness of the tracking can be evaluated by comparing original and motion-stabilized videos and the respective optical maps. It is important to note that, while numerical motion tracking can effectively inhibit motion, it cannot entirely eliminate motion artifacts with strong motion. The baseline in between two action potentials in the corrected (blue and green) optical traces in Figure 7E and Supplementary Figure S4E exhibit residual fluctuations. These (often sinusoidally shaped) fluctuations are residual motion artifacts, which are caused by relative motion between the heart and the light sources in the experimental setup. The relative motion persists even after numerical motion tracking and can principally not be removed with numerical motion tracking and stabilization alone, also refer to Kappadan et al. (20) and Section 4.1. To overcome these residual motion artifacts, it is either necessary to combine numerical motion tracking with ratiometric imaging, refer to Bachtel et al. (44), Zhang et al. (18), and Kappadan et al. (20), or to estimate and correct for the three-dimensional light-field within which the heart moves, refer to Christoph et al. (19).

**Figure 10:**
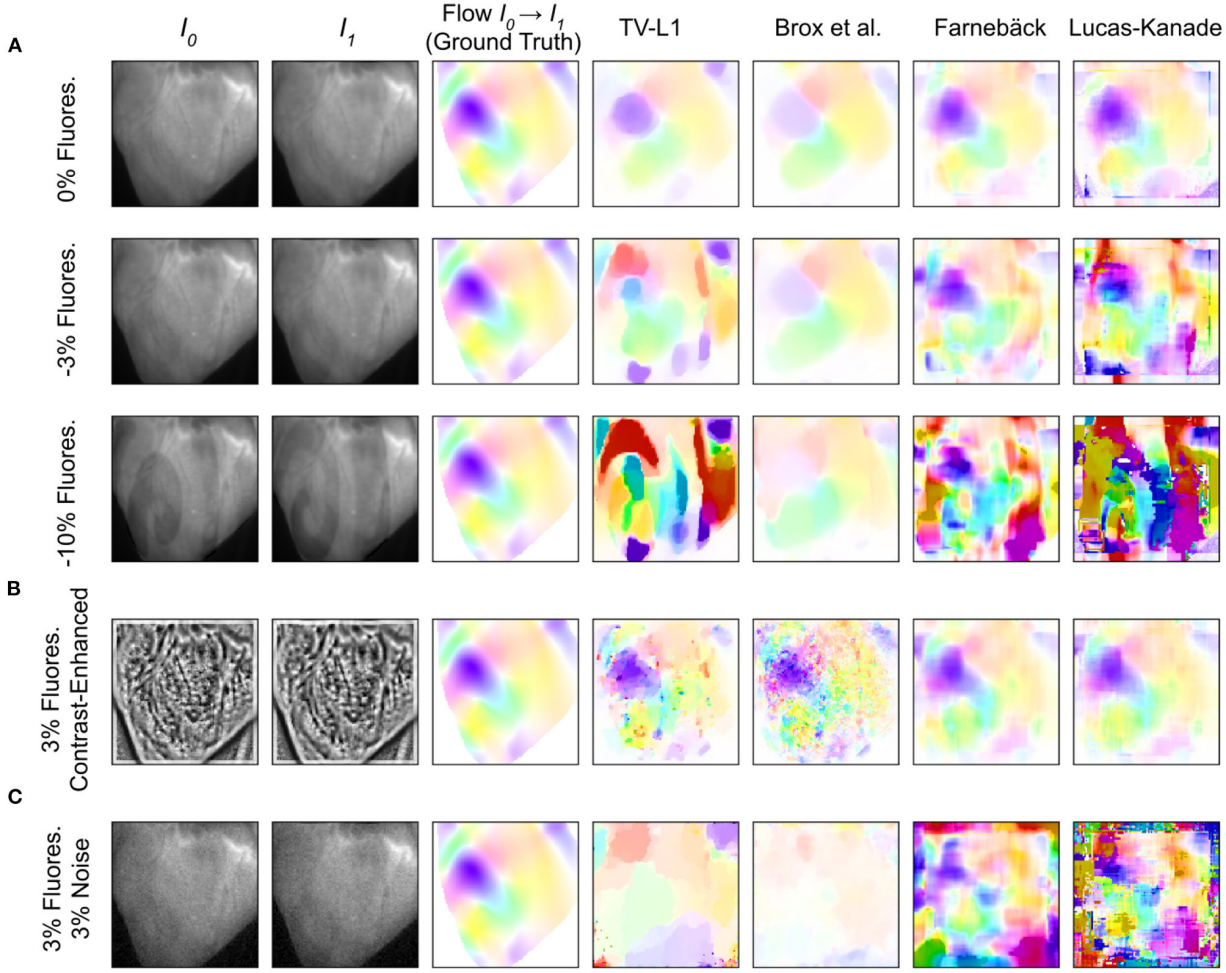
Accuracy of GPU-based motion tracking algorithms applied to synthetic voltage-sensitive optical mapping data. The synthetic data consists of video image pairs showing the tissue in two different deformed states (*I*_0_ and *I*_1_), and dense 2D displacements (hsv-color-code depicts orientation and magnitude, refer to Figure 11) describing the deformation between the two images. In addition, the synthetic video images include action potential wave patterns, which cause a local decrease in fluorescence (−Δ*F*/*F*). The ground truth displacements and tracking outcomes are shown next to each other. **(A)** Tracking accuracy with original video with 0, –3, and –10% fluorescence signal strength, respectively. The increasing fluorescent signal causes tracking artifacts, particularly with the TV-L1 and Lucas-Kanade algorithms. **(B)** Tracking accuracy with contrast-enhanced video images with –3% fluorescence signal strength. The contrast-enhancement reduces tracking artifacts with all algorithms. However, with the TV-L1 and Brox algorithms, the contrast-enhancement generates noisy tracking results (the contrast-enhancement also amplifies noise). **(C)** Tracking accuracy with original video images with 3% noise and –3% fluorescence signal strength. All tracking algorithms are very sensitive to noise: the tracking accuracy deteriorates quickly with noise, refer to Figure 12A.

Comparing the 5 different motion tracking algorithms, we found that the Lucas-Kanade algorithm is very sensitive to fluorescence brightness changes, and the TV-L1 and Farnebäck algorithms are fairly sensitive to these signals, refer to **Figures 10**, **12**. Therefore, it is very likely that these 3 algorithms unintentionally track action potential waves rather than motion with larger fluorescent signal strengths. However, we confirmed that numerical contrast-enhancement as introduced in Christoph and Luther (16) can alleviate these issues. Contrast-enhancement ensures that the algorithms can be used safely with optical mapping videos in conditions with violated brightness constancy caused by fluorescence. Furthermore, we found that both the NVIDIA and Lucas-Kanade algorithms exhibit a severe sensitivity to noise, which likely prohibits their use in most fluorescence imaging applications, refer to **Figure 12**. Overall, we found that it is possible to perform optical mapping experiments with numerical motion tracking and motion artifact compensation on various optical mapping data, and that GPU-acceleration drastically shortens processing times, which allows real-time or high-throughput processing, see next section.

### 3.1. Processing Speed

One of the major practical limitations which we faced in previous optical mapping studies with contracting tissues was the long processing times required for the numerical tracking of the data when using conventional algorithms implemented on the CPU. For example, we used a single-core CPU algorithm implemented in Matlab (45) in Christoph et al. (19), Christoph et al. (6), and Christoph and Luther (16) to track motion and the algorithm required about 10 s per 128 × 128 pixel video frame to compute a motion-compensated version of that frame. By contrast, here, we show that GPU-based implementations in OpenCV can achieve a 5, 000-fold increase in processing speed and can process more than 500 frames per second on the same data. Figures 8, 9 show comparisons of the processing speeds achieved with the TV-L1, Brox, Lucas-Kanade, NVIDIA, and Farnebäck GPU algorithms and the Lucas-Kanade CPU (Matlab) and TV-L1 and Farnebäck CPU (both OpenCV with Python) algorithms when applied to different optical mapping data. All processing speeds were obtained with the NVIDIA RTX 3070 GPU (approx. *$*500). Figure 8 shows a comparison of the processing speeds with low-resolution (128 × 128 pixels) voltage-sensitive optical mapping data, as is also shown on the very right in Figure 3. Figure 8A shows a comparison of the processing speeds of the two slower TV-L1 and Brox algorithms with the reference Matlab CPU implementation of the Lucas-Kanade motion tracking algorithm. While we found that the reference algorithm provides high tracking accuracies (16), it only provided in its particular Matlab implementation processing speeds of about 0.1 fps. By comparison, the OpenCV implementation of the TV-L1 algorithm achieves 1.6 fps on the CPU (running on 1 core) or 4.56 fps on the GPU, respectively, and is, therefore, about 15 times faster on the CPU and about 50 times faster on the GPU than the reference algorithm on the low resolution video data. The Brox motion tracking algorithm achieves processing speeds of 51 fps on the GPU, which is almost 500 times faster than the reference CPU algorithm. The processing speeds of the Brox algorithm and all other faster GPU algorithms cannot be displayed using the same scale on the y-axis as in Figure 8A. They are therefore shown in a separate Figure 8B. The processing times shown in Figures 8A,B include the time required for the pre- (blue) and post-processing (green) of the video data (smoothing, contrast-enhancement, and warping). The processing times were calculated and are stated in milliseconds per frame pair for 128 × 128 pixel video images averaged over 10, 000 video image pairs. The processing speeds of the other faster GPU motion tracking algorithms in Figure 8B are several orders of magnitudes faster than both the TV-L1 and the reference CPU algorithm. Even the Brox algorithm, which is much faster than both the reference and the TV-L1 algorithms and can be barely seen in Figure 8A, is significantly slower than the other algorithms shown in Figure 8B. The GPU-based Lucas-Kanade, Farnebäck, and NVIDIA motion tracking algorithms achieve processing speeds of 795, 852, and 1,000 fps, respectively, when tracking only motion. Together with pre- and post-processing, the effective processing speeds reduce to 489 fps, 510 fps, and 560 fps on 128 × 128 pixel video data, respectively. Since the video data was recorded at 500 fps, the processing can be performed in real-time with the NVIDIA and Farnebäck algorithms. The fast GPU algorithms are about 5, 000 times faster than the CPU reference algorithm used in Christoph et al. (6, 19), Christoph and Luther (16), and Kappadan et al. (20). While the pre- and post-processing significantly contributes to the overall processing times with the fast algorithms shown in Figure 8B, it does not significantly affect the overall processing speeds with the slower tracking algorithms in Figures 8A,B. In Figure 8A the processing time is dominated by the motion tracking, and the pre- and post-processing is negligible in comparison to the time it takes to compute the optical flow fields. Accordingly, with the Brox algorithm, the overall effective processing speed only slightly decreases from 53 fps to 51 fps when taking into account pre- and post-processing. The pre- and post-processing steps are described in more detail in Section 2.4. In Figures 8, 9, we used a kernel diameter size of *k*_*x*_ = *k*_*y*_ = 3 pixels and *k*_*t*_ = 3 time steps for the spatio-temporal smoothing and a kernel diameter size of *k*_*x*_ = *k*_*y*_ = 7 for the contrast-enhancement.

Correspondingly, Figure 9 shows a comparison of the processing speeds with 128 × 128, 256 × 256, 512 × 512, and 1, 024 × 1, 024 pixel video images, respectively. The data shows that GPU-based processing [particularly the 3 fastest algorithms NVIDIA (GPU), Farnebäck (GPU), and Lucas-Kanade (GPU)] provides substantial increases in processing speed, particularly with larger video images. The required processing time scales linearly with the video image size. Figure 9A shows the effective processing speeds including pre- and post-processing and Figure 9B without pre- and post-processing. Figures 8, 9 show that with small video images the Farnebäck CPU algorithm (running on 1 core) is competitive with the GPU-based algorithms (approximately 3x slower). When computing the processing times for the GPU algorithms, the spatio-temporal smoothing was executed on the CPU, while the warping was executed on the GPU. When computing the processing times for the CPU algorithms, all pre- and post-processing was executed on the CPU. Pre- and post-processing on the CPU vs. on the GPU (partially CPU) is about 30% slower and does not lead to a substantial overall reduction in processing speed (for videos with 128 × 128 pixels), also refer to caption in Figure 8. We did not observe a substantial acceleration with the more expensive and more performant RTX 3080 or 3090 GPU models over the RTX 3070 GPU.

In our laboratory, we routinely use numerical motion tracking and motion-stabilization during optical mapping experiments. The high processing speeds allow us to immediately assess the quality of the videos in between recordings. We previously used the Farnebäck GPU algorithm to track motion and compensate for motion artifacts in calcium imaging data of isolated stomach strips (36). The high-resolution calcium imaging data was acquired at a spatial resolution of 1, 280 × 1, 080 pixels and acquisition speeds of 50 fps using a Zyla camera (Andor, Oxford Instruments, UK). With the GPU-based Farnebäck algorithm it became possible to screen and analyze a large number of 15 s long videos containing 750 high-resolution video images, and to effectively reduce motion artifacts and measure calcium transients at high spatial resolutions across large fields of view. Without the high processing speeds, the data analysis would not have been feasible.

### 3.2. Motion Tracking Accuracy

**Figure 11:**
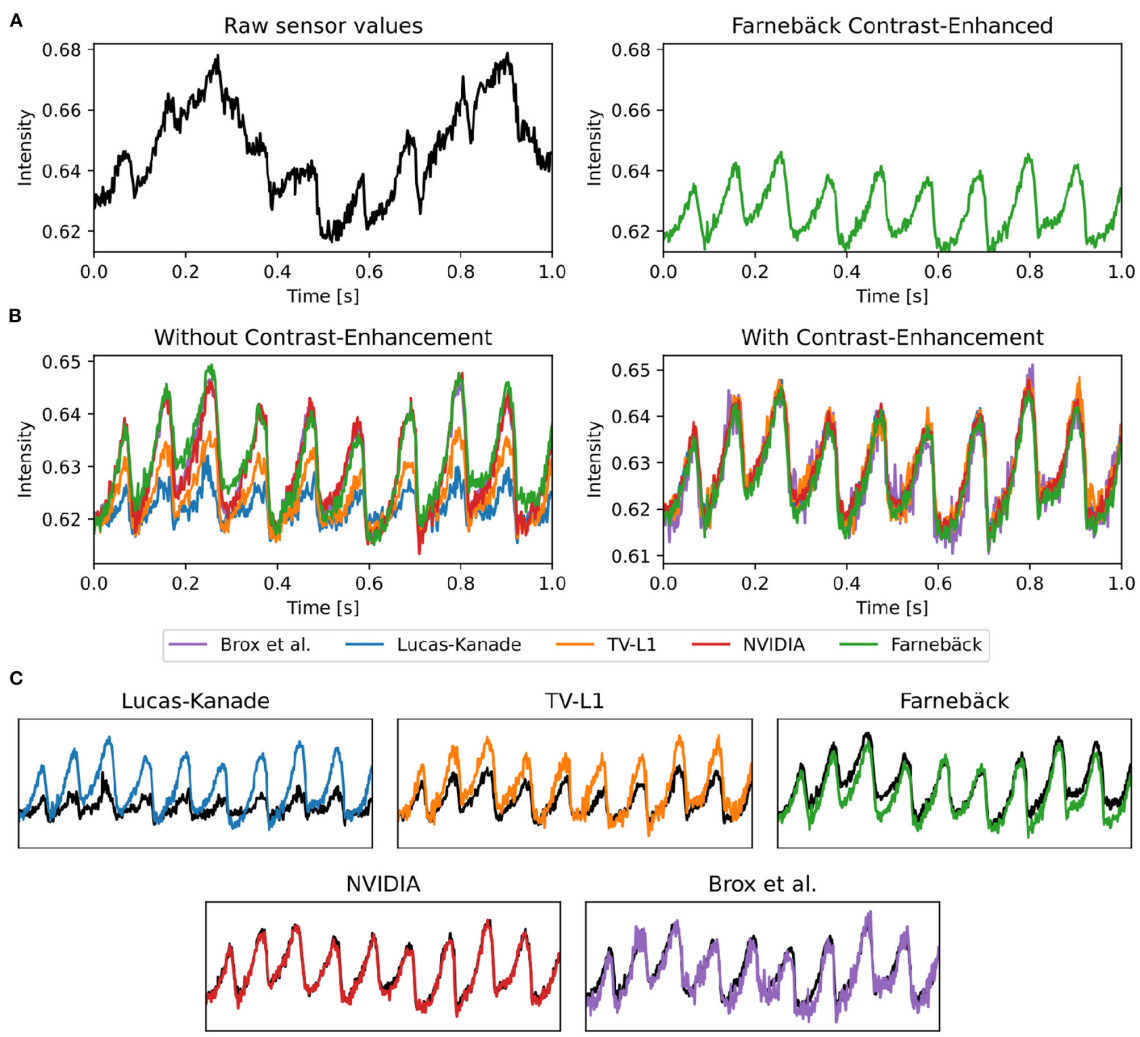
Optical traces obtained from contracting heart surface during ventricular fibrillation (VF) in voltage-sensitive optical mapping recordings before and after numerical motion tracking with 5 different GPU-accelerated motion tracking algorithms (Lucas-Kanade, TV-L1, Farnebäck, NVIDIA, Brox). **(A)** Left: Raw optical trace (black) obtained without numerical motion tracking exhibiting substantial motion artifacts. Right: Optical trace (green) after numerical motion tracking using Farnebäck GPU algorithm and motion-stabilization. The optical trace exhibits a series of action potentials (downstrokes correspond to action potential upstrokes). **(B)** Comparison of numerical motion-stabilization using different GPU algorithms. Left: without contrast-enhancement. Right: with contrast-enhancement. Most of the algorithms are less accurate without contrast-enhancement. With contrast-enhancement, all algorithms behave very similarly and provide similar, sufficiently accurate results. **(C)** Individual comparison of algorithms used with contrast-enhancement (color) and without (black).

**Figure 12:**
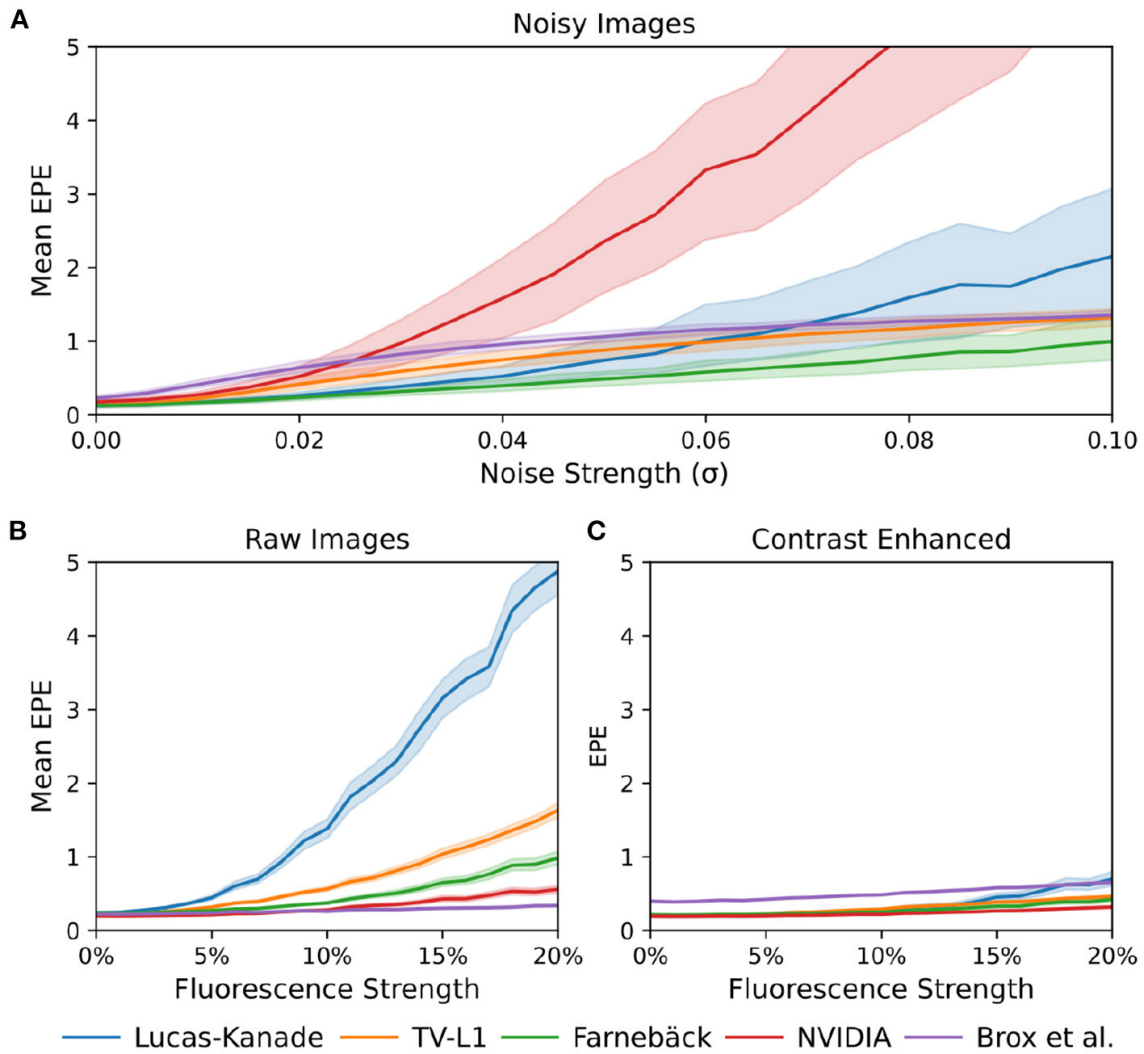
Comparison of motion tracking error of 5 different GPU-accelerated motion tracking algorithms (Lucas-Kanade, TV-L1, Farnebäck, NVIDIA, and Brox). The error was determined using synthetically generated optical mapping recordings with ground truth displacement data obtained in simulations. The error is measured as the average end-to-end point error (EPE) between the estimated (tracked) optical flow vector field and the ground-truth optical flow vector field. **(A)** Tracking error (EPE) increases with noise standard deviation σ. While all algorithms are sensitive to noise, the NVIDIA algorithm is very sensitive to noise and quickly produces larger (than sub-pixel) errors, even at relatively low noise levels. The Farnebäck algorithm is the most robust algorithm against noise. **(B)** Increase in tracking error (EPE error) with increasing fluorescence strength Δ*F*/*F*[%]. **(C)** Compensation for steep increase in tracking (EPE) error with increasing fluorescence strength Δ*F*/*F*[%] when tracking contrast-enhanced versions of the original videos shown in **(B)**.

The tracking accuracy of numerical motion tracking algorithms depends on various factors, such as scene and video image properties, as well as the type, magnitude, and complexity of the motion, among other factors. Here, we determined the effect of fluorescence signal strength, measured as the fractional change in fluorescence intensity Δ*F*/*F*, and image noise σ onto the tracking accuracy using the synthetic video data described in Section 2.1, refer to Figures 10, 12. We found that all tested GPU algorithms are more or less sensitive to and adversely affected by fluorescence and noise. The tracking accuracy deteriorates with increasing fluorescent signal strengths and noise levels, refer to Figures 10A,C, 12A,B and Supplementary Material. However, the first issue related to fluorescent signal strength can be resolved using numerical contrast-enhancement, refer to Figures 10A, 11, 12C. Overall, the Farnebäck algorithm provides the best performance in terms of accuracy and robustness.

When tracking motion in videos of fluorescing and contracting tissue, it is crucial to carefully disentangle motion from physiological phenomena encoded within the fluorescence during the processing. The results are otherwise likely to include both motion and tracking artifacts, as described previously in Christoph et al. (16) and already introduced in Section 1.1. In the absence of any fluorescent signals or vanishing fractional change in fluorescence (Δ*F*/*F* = 0%), refer to top row in Figure 10A, tissue motion simply corresponds to movements of grayvalue patterns (or optical flow) in the image plane. Most tracking algorithms require or assume brightness constancy when computing displacements of tissue regions in between two grayvalue images. However, because fluorescent indicators produce brightness changes when reporting physiological phenomena, brightness constancy is not fulfilled during optical mapping, see second and third row in Figure 10A. For example, during the depolarization phase of the action potential the fluorescent dye Di-4-ANEPPS produces a decrease in fluorescence measured behind a near-infrared long-pass filter (because the emission spectrum shifts toward shorter wavelengths during the depolarization). Therefore, action potential waves correspond to grayvalue patterns moving across another moving and deforming grayvalue pattern representing the movements of the tissue, refer to the two left video images in the third row in Figure 10A with Δ*F*/*F* = −10%. In other words, action potential, or calcium waves produce optical flow just as much as movements of the tissue. As a result, tracking algorithms can inadvertently track either or a mix of the two phenomena. This effect was previously described in Christoph and Luther (16) and can be observed when examining the tracking outcome with the TV-L1, Farnebäck, and Lucas-Kanade algorithms in the second and third rows in Figure 10A. Without any fluorescent signal (|Δ*F*/*F*| = 0%), all algorithms track the ground truth flow fields sufficiently accurate, refer to the first row in Figure 10A and the left region in the graph shown in Figure 12B, which shows the average EPE of each algorithm plotted over fluorescence strength from 0 to 20% absolute fractional change in fluorescence |Δ*F*/*F*|. The EPE was determined over a dataset containing 20, 000 image pairs. All algorithms exhibit small, sub-pixel tracking errors close to zero at 0% fractional change in fluorescence. However, the EPEs grow quickly with increasing fluorescent strengths: the Lucas-Kanade, TV-L1, and Farnebäck algorithms exceed EPEs larger than 1 pixel with fluorescence strengths above 5, 10, and 20%, respectively. Correspondingly, the 2nd and 3rd rows in Figure 10A show how the tracked flow fields become less accurate or deteriorate entirely with 3 and 10% fluorescent signal strengths. With the TV-L1 algorithm, the resulting displacement vectors correlate with the shape of the action potential conduction pathway, suggesting that the algorithm inadvertently tracks the action potential. The Brox algorithm is an exception, as it does not appear to be affected by strong fluorescent signals. However, the Brox algorithm produces (too small) displacements, which do not seem to describe the motion of the tissue accurately.

**Figure 13:**
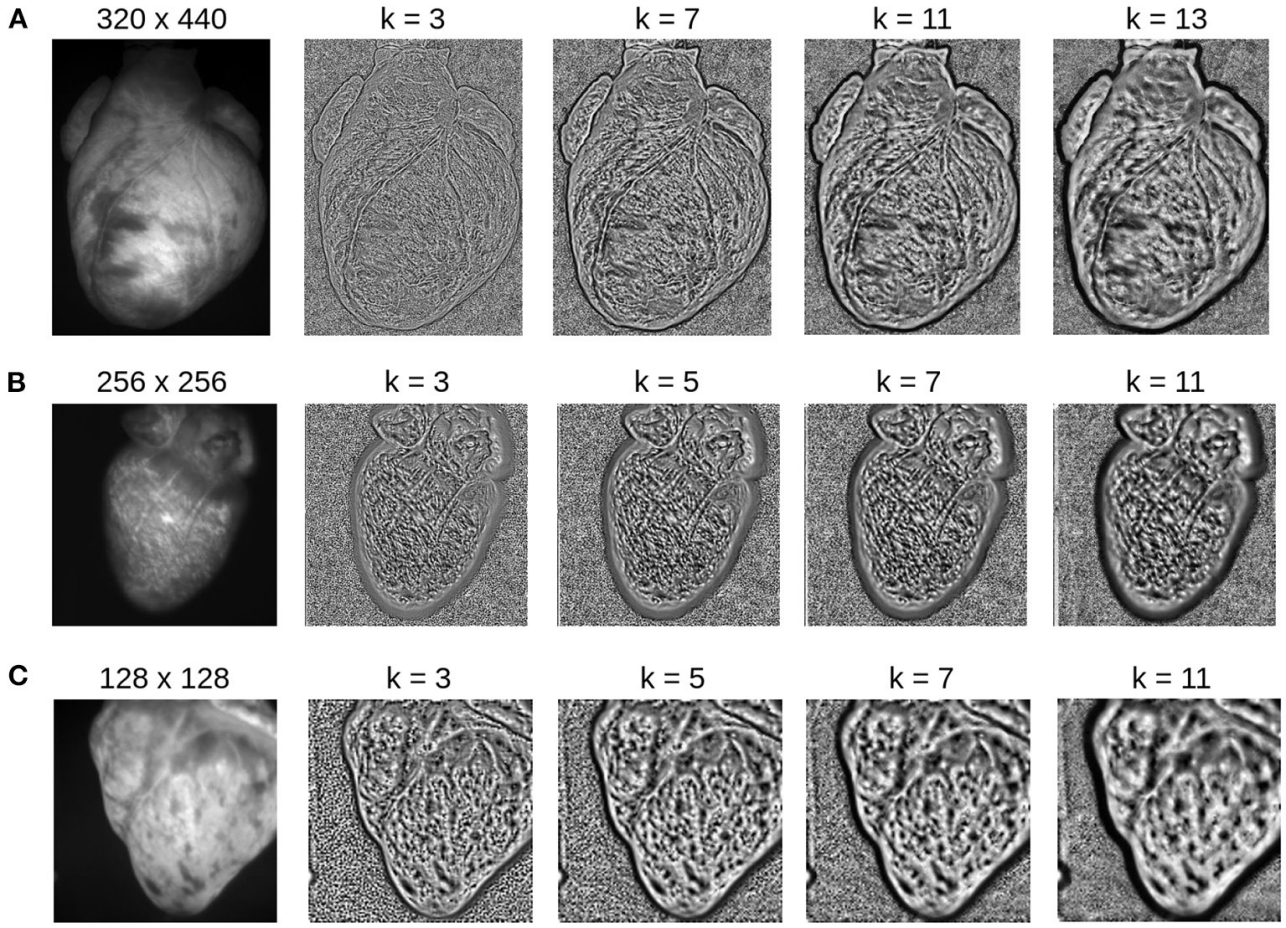
Contrast-enhancement with different kernel diameters *k* for three different optical mapping recordings. The kernel size is an important input parameter, which needs to be adjusted to match the properties of the video image. **(A)** Contrast-enhancement with 320 × 440 video images shown in Figure 4: the ideal kernel diameter for the contrast-enhancement is about 9 or 11 pixels. **(B)** Contrast-enhancement with 256 × 256 video image: the ideal kernel diameter is about 7 pixels. **(C)** Contrast-enhancement with 128 × 128 video image: the ideal kernel diameter is about 3 or 5 pixels. A too small kernel creates noise, whereas a too large kernel reduces the local contrast, enhances large scale gradients, and/or produces too large features.

The accidental tracking of action potential or calcium waves can be minimized by generating contrast-enhanced versions of the original videos, as described in Section 2 and previously in Christoph and Luther (16) and shown in Figures 10B, 11B. In short, the contrast-enhanced videos are calculated by computing the maximal and minimal pixel intensities within a small circular region around each pixel and normalizing that pixel by these two values. As a result, local grayvalue patterns representing the tissue become amplified yielding videos with maximal spatial contrast (contrast-enhancement acts as a spatial high-pass filter while it suppresses temporal fluctuations). At the same time, time-varying signals caused by fluorescent reporters are inhibited in contrast-enhanced videos. The video images in Figure 10B were calculated using kernels with a diameter of *k*_*x*_ = *k*_*y*_ = 11 pixels, also refer to Figure 13 for a comparison of contrast-enhanced videos with different kernel sizes. As Figure 10B and the plots in Figure 12C show, all GPU algorithms produce more reliable tracking results with contrast-enhancement, and the overall EPE becomes substantially reduced with strong fluorescent signals. Mismatches between tracked and ground-truth displacement vectors stay below sub-pixel levels for all algorithms and all fluorescence strengths with contrast-enhancement. In particular, the Farnebäck and Lucas-Kanade algorithms achieve tracking accuracies comparable to when tracking the original videos without fluorescent signals (|Δ*F*/*F* = 0%|), c.f. row 1 in Figure 12A. Overall, it appears that contrast-enhancement is a viable approach that works with many different numerical motion tracking algorithms. However, we found that contrast-enhancement can cause noisier tracking outcomes, particularly with the TV-L1 and Brox algorithms, refer to Figures 10B, 11B,C. We accordingly smoothed the tracked displacement vector fields when applying the algorithms to contrast-enhanced video data.

The sensitivity of the motion tracking algorithms to noise is shown in Figures 10C, 12A. The Gaussian noise with zero mean and standard deviation σ was added to each pixel of each synthetic video image individually. The amount of noise strength of 3% shown in Figure 10C corresponds to noise with a standard deviation of σ = 0.03 intensity counts added or subtracted on average to or from each pixel. Note that the videos are normalized and contain only pixels with intensity values *I*(*x, y*)∈[0, 1]. The plots in Figure 12A show how the tracking accuracy of the Lucas-Kanade, TV-L1, Farnebäck, NVIDIA, and Brox algorithms deteriorates with increasing noise strengths. The EPE was determined over a dataset containing 20, 000 image pairs. The NVIDIA and Lucas-Kanade algorithms are the most sensitive to noise: their mean EPE increases steeply with noise levels σ>0.02−0.04. The TV-L1, Brox, and Farnebäck algorithms are more robust, but their accuracy also degrades significantly with noise. Overall, the Farnebäck algorithm performed best with noise, particularly with noise levels below σ < 0.06, and provides sub-pixel accuracies up to noise levels of σ = 0.08.

## 4. Discussion

The ability to image electrophysiological and mechanical phenomena in contracting tissues simultaneously at high speeds and spatial resolutions is a highly sought-after methodology in physiological research. Optical techniques, such as optical mapping, are in principle ideally suited for this task. However, performing optical mapping with moving tissues is still challenging. In this study, we addressed one of the key hurdles in the application of numerical motion tracking to optical mapping data: identifying motion tracking algorithms that i) are applicable to optical mapping data and ii) provide sufficiently high processing speeds. We demonstrated that freely-available, open-source motion tracking algorithms implemented on a GPU can be used to track motion in various types of optical mapping data while they also provide substantial increases in processing speeds. High processing speeds are necessary as they open the path for the routine, day-to-day application of numerical motion tracking in optical mapping experiments, open the path for real-time applications, and will allow the high-throughput offline analysis of large video data sets as demonstrated in Vogt et al. (36). In previous studies (6, 16, 19), computations were so time consuming that they prohibited a more systematic and streamlined analysis of optical mapping videos. Now we routinely compensate motion artifacts fully automatically and are able to analyze motion-compensated optical maps on the fly during an optical mapping experiment in our laboratory. The data in this study represent typical use cases, for which we show that numerical motion artifact compensation can be performed efficiently suggesting that the methodology could be used routinely by other laboratories. Real-time processing could be utilized to measure and monitor both electrophysiological and mechanical quantities instantaneously, to eventually control experimental parameters in response to the real-time monitoring. It could, furthermore, obviate time-consuming post-processing and the storing of the video data for post-processing. In summary, we demonstrated that with GPU-based numerical motion artifact compensation it is now possible to perform optical mapping with contracting cardiac tissue with any video camera (with low-cost high-resolution cameras such as the IDS or Basler cameras or more expensive medium- to high-resolution cameras such as the Micam N256 or the Andor Zyla cameras, respectively). The high processing rates can be achieved with consumer GPU hardware that costs about *$*500 (e.g., NVIDIA Geforce RTX 3070 GPU), which could facilitate the more widespread adoption of numerical motion tracking and motion stabilization in basic cardiovascular research.

Overall, the Farnebäck GPU algorithm is a suitable candidate for tracking motion in optical mapping videos. It provides high processing speeds while being only mildly sensitive to noise and fluorescence artifacts, refer to Figure 12. Among the tested algorithms, we found it to be the most robust algorithm able to handle also large deformations during sinus rhythm. It should be applicable and provide sufficient tracking accuracies in a wide range of optical mapping applications. While the GPU implementations of the Lucas-Kanade and NVIDIA motion tracking algorithms provide similarly high processing rates, they are extremely sensitive to noise, which may limit them to low-noise applications. Furthermore, the Lucas-Kanade algorithm is very sensitive to the fluorescent signal. The Brox algorithm, by comparison, behaves very differently: it is much slower, is robust against fluorescence artifacts, but produces noisy vector fields when tracking contrast-enhanced videos and consistently underestimates motion in the 128 × 128 pixel video images. Surprisingly, the Lucas-Kanade algorithm is the least robust, most sensitive algorithm of the 5 GPU algorithms. In previous studies (6, 16, 19), we used a CPU variant of the Lucas-Kanade algorithm (45) and achieved high tracking accuracies, which we validated in the same way as in this study with synthetic data. This discrepancy may be related to differences in the implementations of the algorithm ((45) vs. OpenCV). Furthermore, we used low-light optics and cameras (Teledyne Photometrics Evolve camera) and paid much attention to properly staining and illuminating the tissue to achieve high signal-to-noise ratios. Additionally, we smoothed the video data (using small kernels in the order of 3–5 pixels in diameter) before tracking to suppress residual noise. Therefore, with 5–10% fluorescent signal strength (Δ*F*/*F*) and contrast-enhancement, we operated in a regime shown on the left in Figure 12C, where the Lucas-Kanade algorithm still provides high accuracies. Our study highlights the importance of the pre-processing (spatio-temporal smoothing) of the video data and confirms the effectiveness of contrast-enhancement to increase the robustness of the tracking. The severe sensitivity of some of the GPU motion tracking algorithms to noise is yet a major limitation, which needs to be addressed in future research.

While there is an abundance of other numerical motion tracking algorithms, we focused on some of the most popular and freely available motion tracking algorithms to establish an overview of the applicability of these 'classical' algorithms to optical mapping data. The 5 motion tracking algorithms we tested are part of OpenCV, an open-source computer vision library, which is freely available on all major operating systems. Therefore, it should be possible to reproduce our findings and use them as a reference and guidance when developing our own motion tracking software. We tested all algorithms with their default parameters and there may be other parameter regimes that may yet improve the performance of the algorithms. In future research, we aim to research other motion tracking techniques and develop custom motion tracking algorithms, which are specifically designed to be used in optical mapping studies.

### 4.1. Limitation: Numerical Motion Compensation Alone Does Not Abolish Motion Artifacts

In this study, we focused on assessing the performance of different motion tracking algorithms and showed that motion tracking can track and stabilize motion in optical mapping videos accurately and can significantly reduce motion artifacts. However, motion tracking alone cannot entirely remove motion artifacts *per se*. With motion tracking, the optical measurement can be performed in a co-moving frame of reference. This means that the frame of reference is changed from the static laboratory coordinate-based frame measuring the activity pixel-by-pixel to the co-moving frame, in which the motion of the heart is apparently non-existent. However, the relative motion between the (moving) heart and the (static) light sources illuminating the heart is not physically removed by the tracking. Even though the heart's motion is no longer visible in the motion-compensated optical maps, the relative motion persists after motion compensation. Therefore, as the frame of reference changes from static to co-moving, the heart appears to stop moving and instead there is apparent movement in the light sources, refer to Supplementary Videos 1, 3, 4. The continuing movement of the heart relative to the light sources causes motion artifact. These residual motion artifacts can be seen in Figures 4E, 7E and, most intuitively, in Supplementary Figure S4E: the green sinusoidally shaped baseline reflects the back and forth motion of the heart between brighter and darker illuminated areas. Illumination-related motion artifacts can only be inhibited by combining numerical motion tracking with either i) ratiometric imaging (18, 20, 44), or ii) estimating the motion of the heart in a three-dimensional inhomogeneous light-field and correcting the optical signals for this change in illumination (19). Illumination-related motion artifacts are most critical during sinus rhythm, refer to Figures 4, 7, and less critical during fibrillation, as the illumination varies more strongly with larger amplitudes of motion, also refer to Figure 12 in Kappadan et al. (20). There are other potential sources for motion artifacts including absorption and reflection changes due to the orientation and compression state of the tissue. However, such effects have never been described in detail.

To date, we must conclude that optical mapping with contracting cardiac tissues remains challenging. Not all measurements which can be performed with pharmacologically contraction-inhibited hearts can equally be performed as easily with contracting hearts. For instance, while it is fair to compute activation maps from sinus rhythm data as shown in Figure 4C using only numerical motion compensation, or compute dominant frequencies or phase maps from ventricular fibrillation data as shown in Figure 5E (with the Farnebäck algorithm) using only numerical motion compensation, it may be necessary to combine numerical motion compensation with ratiometry or numerical light-field correction in order to measure action potential durations accurately from a strongly contracting heart. The future of electromechanical optical mapping is bright. However, further study is necessary until it becomes a widely applicable and easy-to-use imaging technique.

## 5. Conclusion

We demonstrated that it is possible to perform optical mapping with contracting cardiac tissues in real-time and with large video images using GPU-accelerated numerical motion tracking and motion compensation algorithms. We tested 5 different motion tracking algorithms on optical mapping data of contracting rabbit, mouse, and pig hearts and cardiac cell cultures imaged with 4 different high-speed cameras as well as on synthetic optical mapping data. We found that the different motion tracking algorithms can behave very differently. Among the tested algorithms, the Farnebäck GPU algorithm is one of the fastest and best suited algorithms for optical mapping data providing sufficiently high tracking accuracies and very high processing speeds. We achieved real-time processing speeds with small videos (128 × 128 pixels) acquired at 500 fps and with large videos (800 × 800 pixels) acquired at 40 fps. The high processing speeds provided by GPU-based algorithms could make electromechanical optical mapping more attractive for routine, day-to-day use and open the path for real-time or high-throughput applications.

## Data Availability Statement

The data supporting the conclusions of this article will be made available by the authors, upon reasonable request.

## Ethics Statement

The experiments in this study were reviewed and approved by the Institutional Animal Care and Use Committee (IACUC) at the University of California, San Francisco.

## Author Contributions

JL created the synthetic data and implemented and evaluated the algorithms. GK produced the cell culture. JL, NR, and JC conducted the imaging experiments. JL and JC designed the figures. JL, NR, GK, and JC discussed the results and wrote the manuscript. All authors contributed to the article and approved the submitted version.

## Funding

This research was funded by the University of California, San Francisco (to JC), the German Center for Cardiovascular Research (DZHK e.V.), partnersite Göttingen (to GK and JC), the Sandler Foundation (to JC), and by the German Federal Ministry of Education and Research (BMBF) – German Network of RASopathy Research (GeNeRARe, grant number: 01GM1902D, to GK). NR was a research Fellow supported by the Sarnoff Cardiovascular Research Foundation.

## Conflict of Interest

The authors declare that the research was conducted in the absence of any commercial or financial relationships that could be construed as a potential conflict of interest.

## Publisher's Note

All claims expressed in this article are solely those of the authors and do not necessarily represent those of their affiliated organizations, or those of the publisher, the editors and the reviewers. Any product that may be evaluated in this article, or claim that may be made by its manufacturer, is not guaranteed or endorsed by the publisher.

## Footnotes

^1^ https://developer.nvidia.com/opticalflow-sdk

^2^ https://developer.nvidia.com/optical-flow-sdk-source-code
