# Supplementary material for: Real-Time Optical Mapping of Contracting Cardiac Tissues With GPU-Accelerated Numerical Motion Tracking
Source: Front Cardiovasc Med. 2022 May 24;9:787627. doi: 10.3389/fcvm.2022.787627 (PMC9172765; doi:10.3389/fcvm.2022.787627)
Supplement: Supplementary file 10 [file Data_Sheet_1.pdf]

Supplementary Material

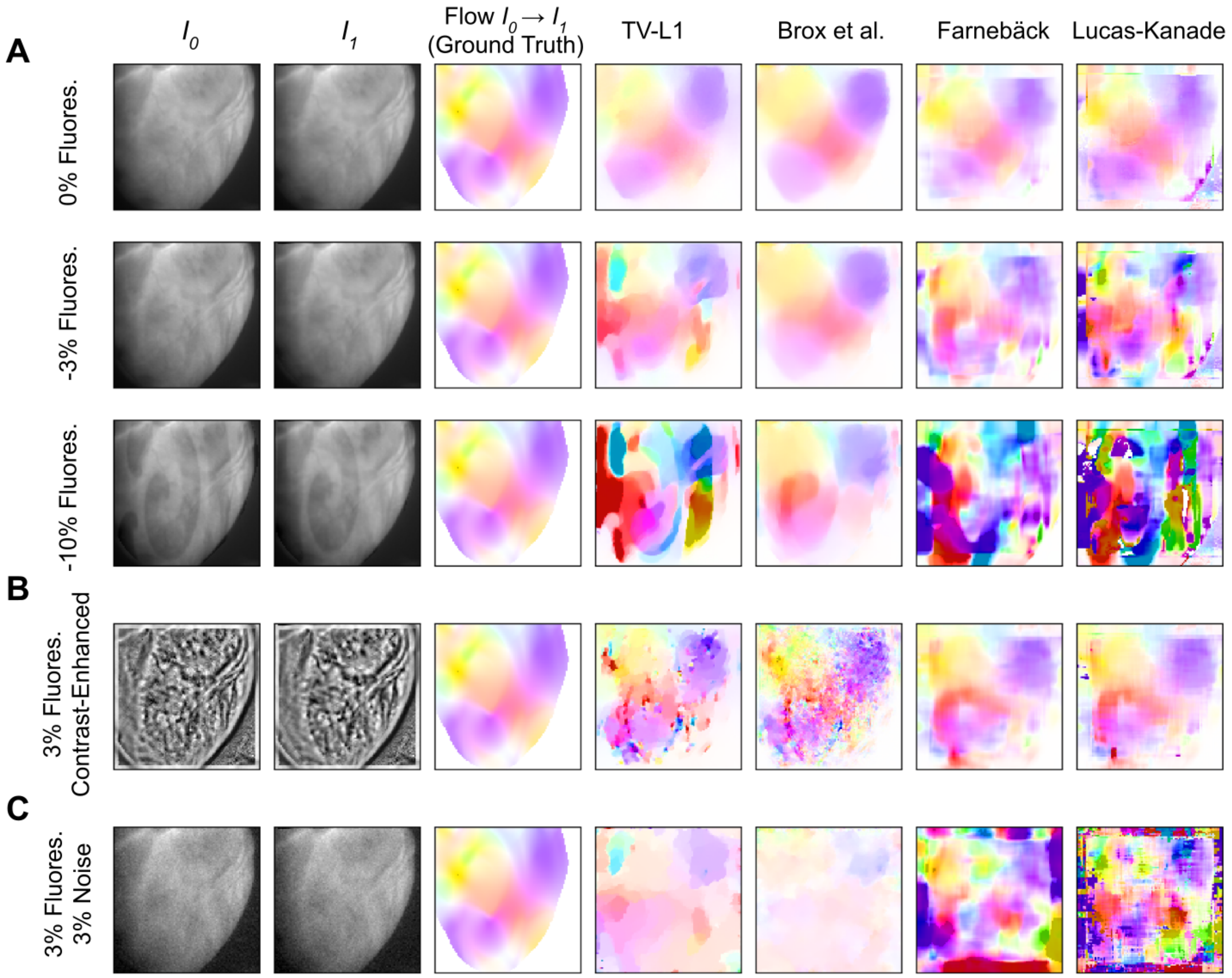

Figure S1. Accuracy of GPU-based motion tracking algorithms applied to synthetic voltage-sensitive optical mapping data. The synthetic data consists of video image pairs showing the tissue in two different deformed states (I_0 and I_1), and dense 2D displacement data describing the deformation between the two images. In addition, the synthetic video images contain action potential wave patterns, which cause a local decrease in fluorescence strength ($-\Delta F/F$). The ground truth displacements and tracking outcome are color-coded (orientation and magnitude). **A)** Tracking accuracy with original video images with 0%, 3% and 10% fluorescence signal strength, respectively. The increasing fluorescent signal causes tracking artifacts, particularly with the TV-L1 and Lucas Kanade algorithms. **B)** Tracking accuracy with contrast-enhanced video images with 3% fluorescence signal strength. The contrast-enhancement reduces tracking artifacts with all algorithms. However, with the TV-L1 and Brox algorithms the contrast-enhancement generates noisy tracking results. **C)** Tracking accuracy with original video images with 3% noise and 3% fluorescence signal strength.

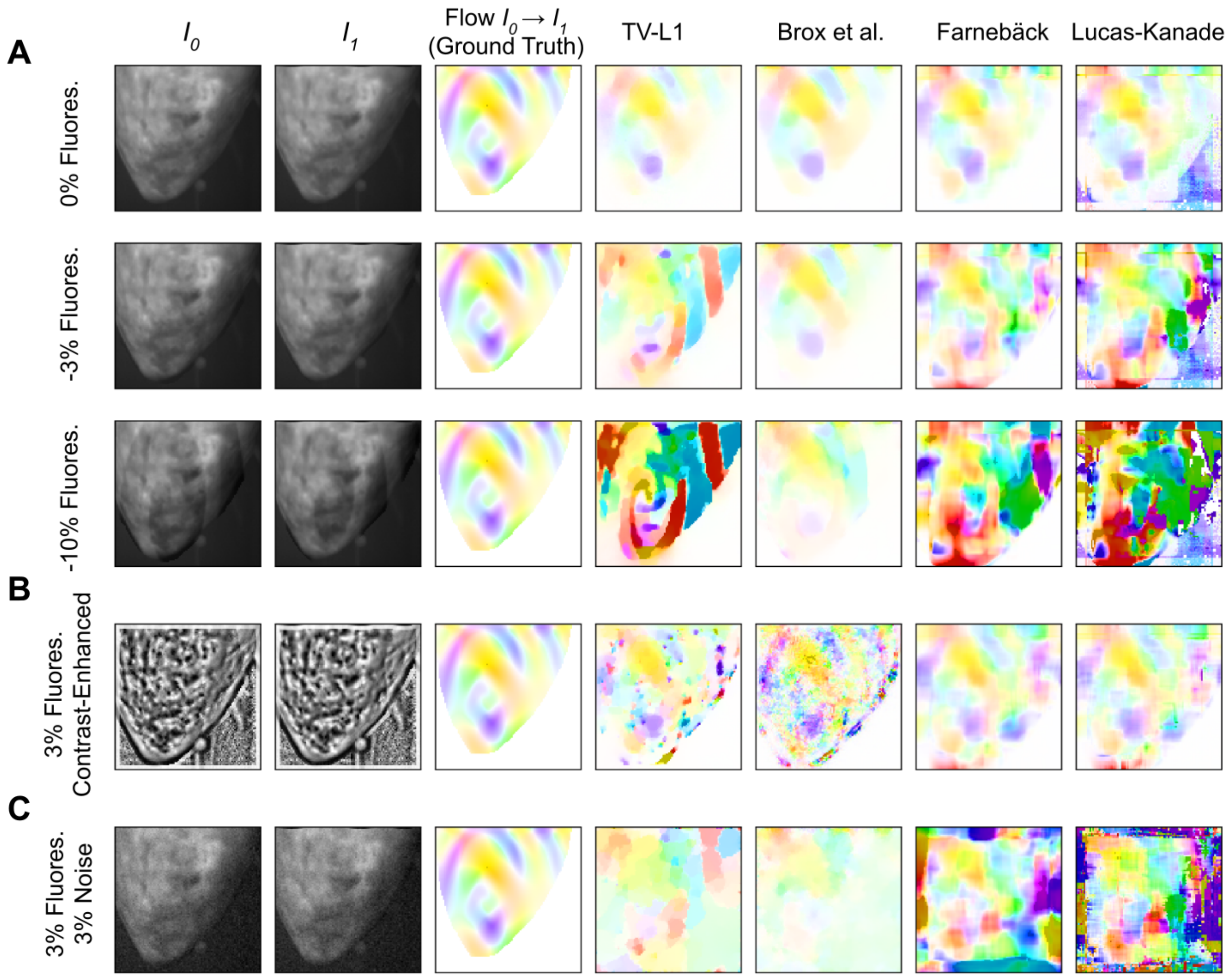

Figure S2. Accuracy of GPU-based motion tracking algorithms applied to synthetic voltage-sensitive optical mapping data. The synthetic data consists of video image pairs showing the tissue in two different deformed states (I_0 and I_1), and dense 2D displacement data describing the deformation between the two images. In addition, the synthetic video images contain action potential wave patterns, which cause a local decrease in fluorescence strength ($-\Delta F/F$). The ground truth displacements and tracking outcome are color-coded (orientation and magnitude). **A)** Tracking accuracy with original video images with 0%, 3% and 10% fluorescence signal strength, respectively. The increasing fluorescent signal causes tracking artifacts, particularly with the TV-L1 and Lucas Kanade algorithms. **B)** Tracking accuracy with contrast-enhanced video images with 3% fluorescence signal strength. The contrast-enhancement reduces tracking artifacts with all algorithms. However, with the TV-L1 and Brox algorithms the contrast-enhancement generates noisy tracking results. **C)** Tracking accuracy with original video images with 3% noise and 3% fluorescence signal strength.

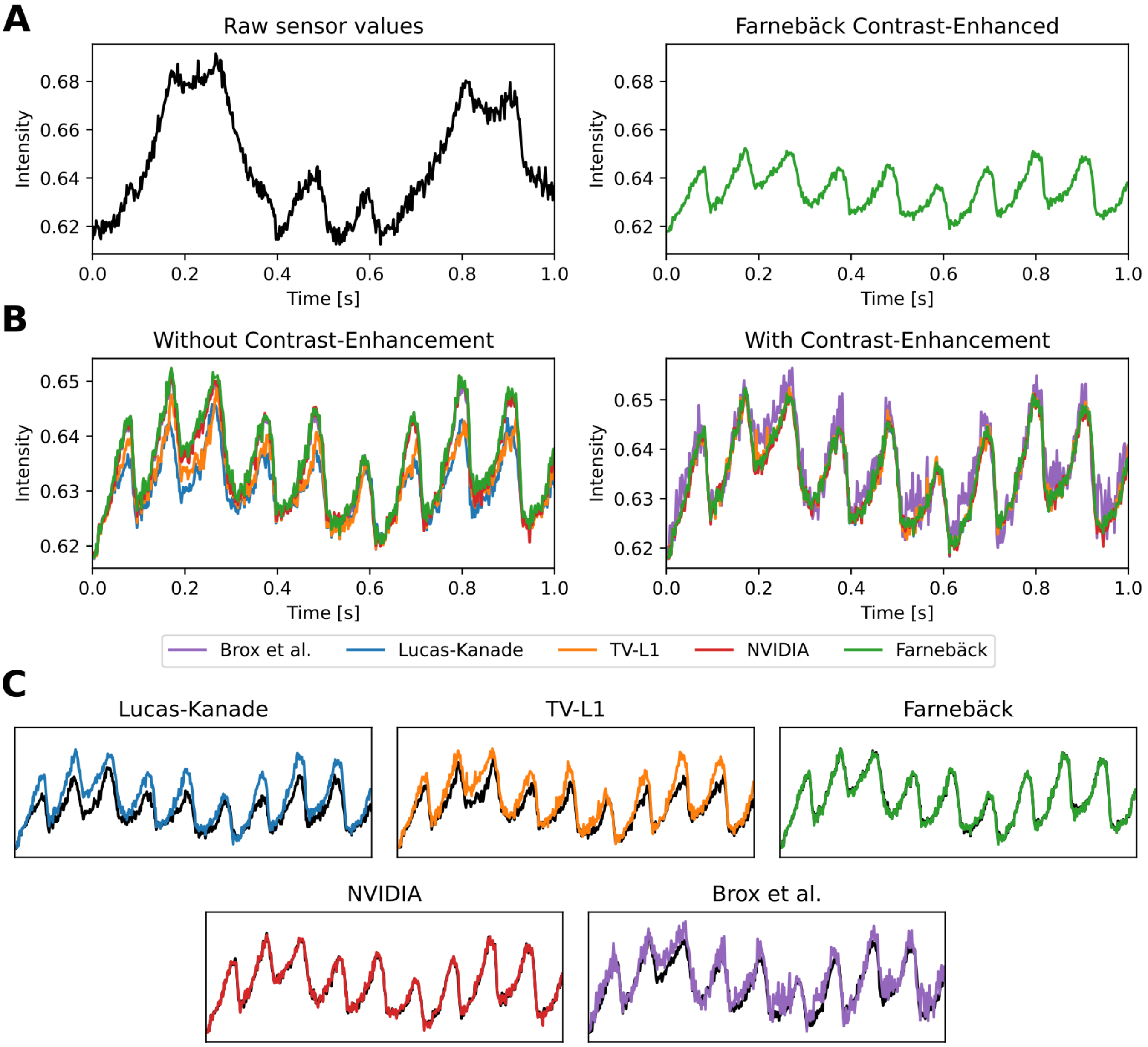

Figure S3. Optical traces obtained from contracting heart surface in voltage-sensitive optical mapping recordings of ventricular fibrillation before and after numerical motion tracking with 5 different GPU-accelerated motion tracking algorithms (Lucas-Kanade, TV-L1, Farnebäck, NVIDIA, Brox). **A)** Left: Raw optical trace (black) obtained without numerical motion tracking exhibiting substantial motion artifacts. Right: Optical trace after numerical motion tracking using Farnebäck GPU algorithm and motion-stabilization exhibiting a series of action potentials (downstrokes correspond to action potential upstrokes). **B)** Comparison of numerical motion-stabilization using different GPU algorithms. Left: without contrast-enhancement. Right: with contrast-enhancement. Without contrast-enhancement most of the algorithms perform less accurate. With contrast-enhancement all algorithms provide similar and sufficiently accurate results. **C)** Individual comparison of performance of tracking algorithms and resulting motion-stabilization when tracking videos with contrast-enhancement (color) and without (black).

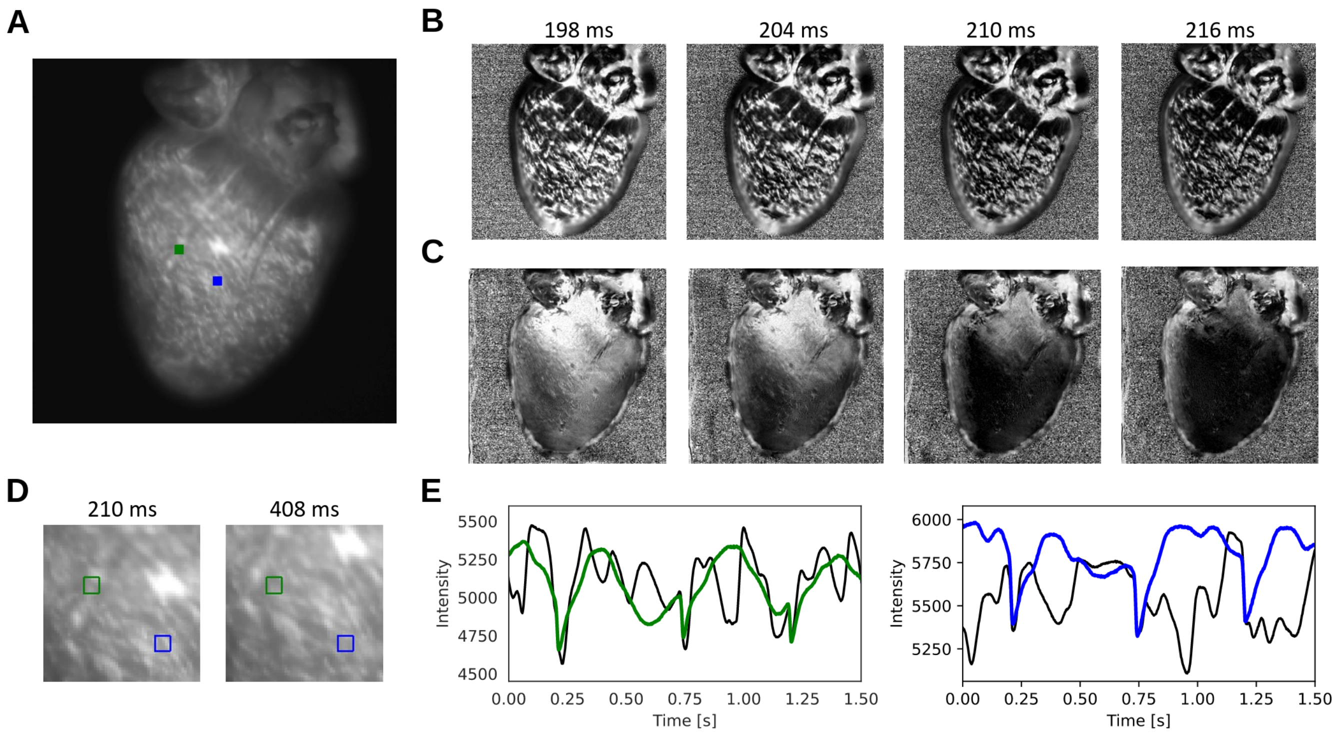

Figure S4. Numerical motion tracking and motion artifact compensation performed with Farnebäck GPU motion tracking algorithm with optical mapping video of rabbit heart during sinus rhythm recorded with a Brainvision Scimedia MiCAM N256 camera with voltage sensitive-staining (Di-4-ANEPPS), see also Supplementary Video 9. **A)** Cropped video image (200×200 pixels) from original recording (500fps, 256×256 pixels). ROI (green and blue, 6×6 pixels) used to extract optical traces shown in E). **B)** Motion and deformation of heart surface around ROIs shown in (A) over time (50×50 pixels). **C)** Pixel-wise normalized video without numerical motion compensation and with motion artifacts. **D)** Pixel-wise normalized video after numerical motion tracking and compensation and with significantly reduced motion artifacts. The action potential wave is visible as a dark wave. **E)** Optical traces obtained from videos before (black) and after (green and blue) motion tracking and artifact compensation. Traces averaged over the two rectangular regions shown in A).

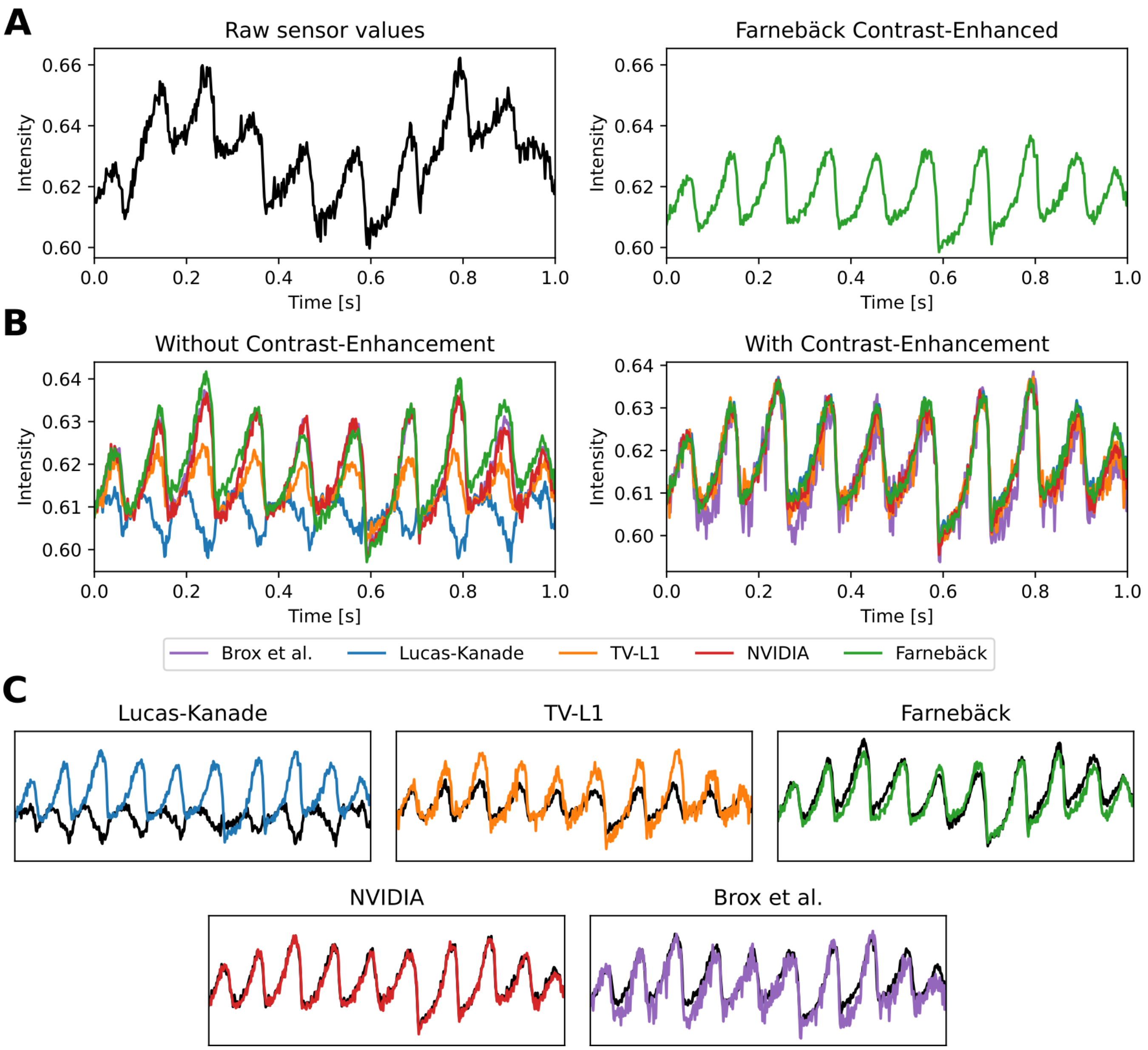

Figure S5. Optical traces obtained from contracting heart surface in voltage-sensitive optical mapping recordings of ventricular fibrillation before and after numerical motion tracking with 5 different GPU-accelerated motion tracking algorithms (Lucas-Kanade, TV-L1, Farnebäck, NVIDIA, Brox). **A)** Left: Raw optical trace (black) obtained without numerical motion tracking exhibiting substantial motion artifacts. Right: Optical trace after numerical motion tracking using Farnebäck GPU algorithm and motion-stabilization exhibiting a series of action potentials (downstrokes correspond to action potential upstrokes). **B)** Comparison of numerical motion-stabilization using different GPU algorithms. Left: without contrast-enhancement. Right: with contrast-enhancement. Without contrast-enhancement most of the algorithms perform less accurate. With contrast-enhancement all algorithms provide similar and sufficiently accurate results. **C)** Individual comparison of performance of tracking algorithms and resulting motion-stabilization when tracking videos with contrast-enhancement (color) and without (black).

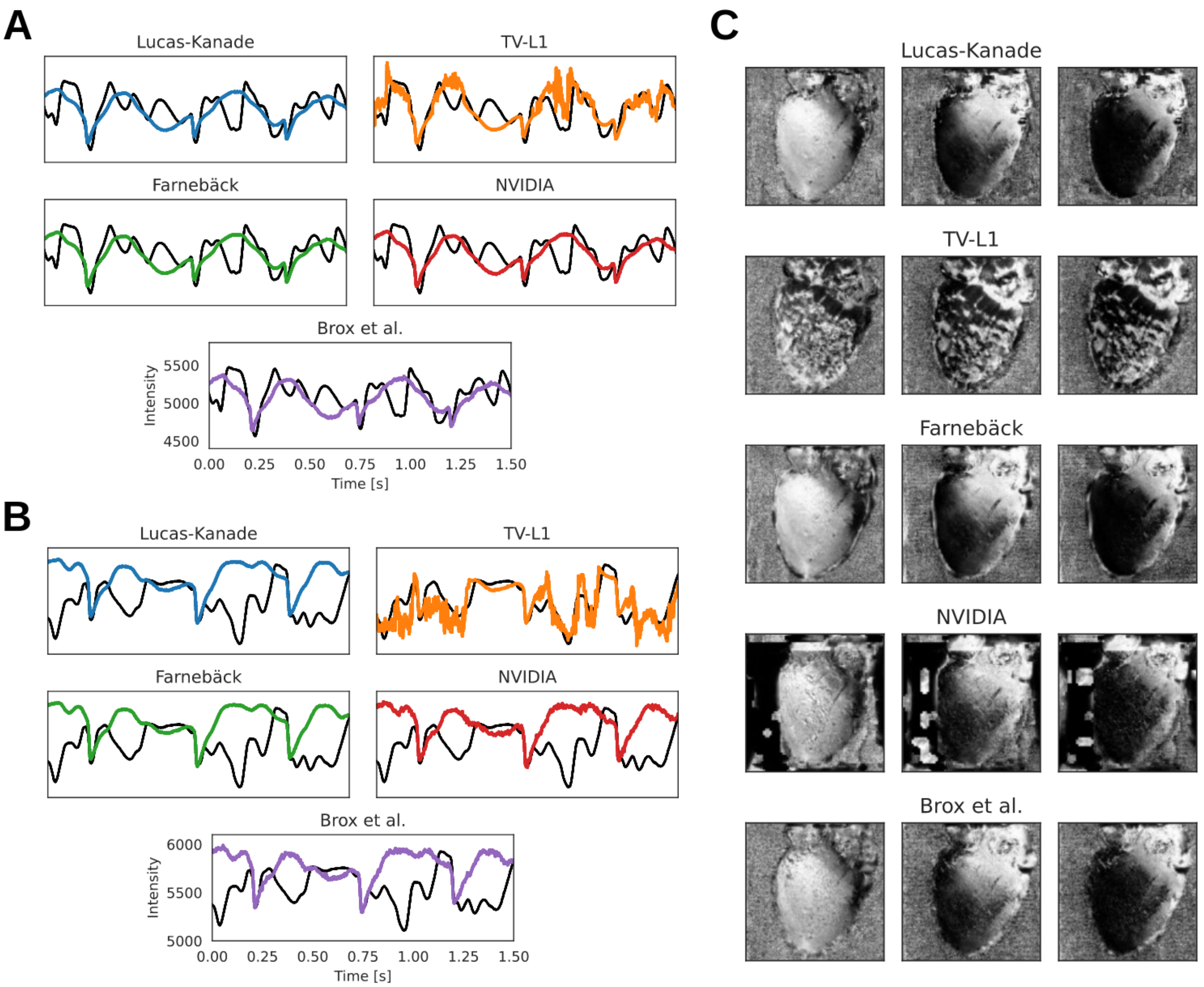

Figure S6. Comparison of motion-stabilized optical traces and optical maps obtained with 5 different GPU-based motion tracking algorithms imaging a beating rabbit heart during sinus rhythm with a Brainvision Scimedia MiCAM N256 camera and voltage-sensitive fluorescent dye (Di-4-ANEPPS), see also Fig. S4 and Supplementary Video 9. **A)** Optical traces obtained from the left (green) site shown in Fig. S4A,D), before (black) and after (colored) motion tracking and artifact compensation, c.f. left (green) trace in E). Residual illumination-related ,otion artifacts **B)** Optical traces obtained from the right (blue) site shown in Fig. S4A,D), before (black) and after (colored) motion tracking and artifact compensation, c.f. right (blue) trace in E). **C)** Optical maps (pixel-wise normalized) showing effectiveness and ineffectiveness of different algorithms in producing motion-stabilized videos. With some algorithms, the action potential waves are superimposed by motion and other artifacts (TV-L1, NVIDIA). All optical maps contain residual illumination-related motion artifacts.

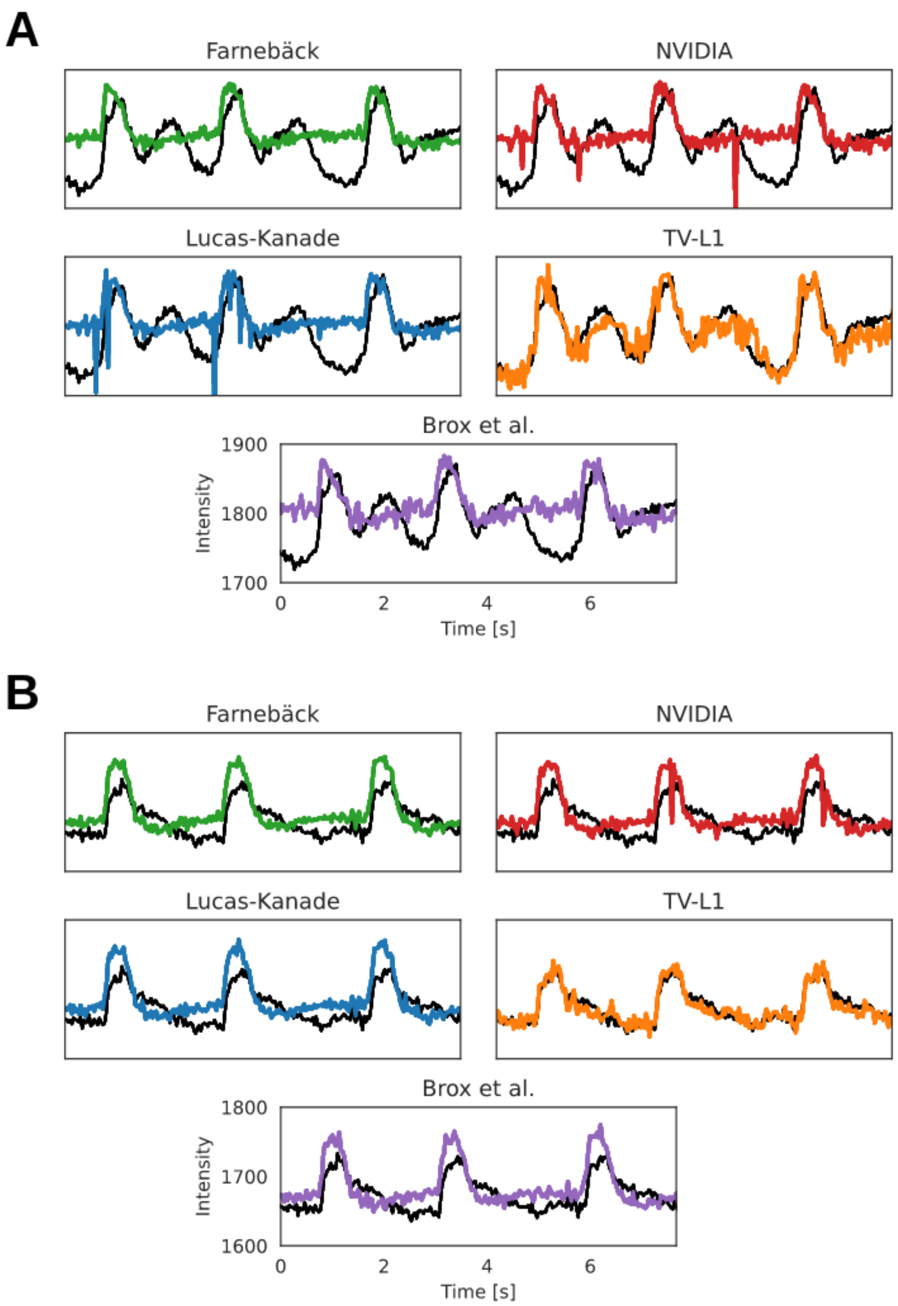

Figure S7. Comparison of motion-stabilized optical traces and optical maps obtained with 5 different GPU-based motion tracking algorithms imaging a strongly contracting and deforming cardiac cell culture (derived from hiPSC-CMs) with a IDS μEye UI-3060CP-M-GL camera and voltage-sensitive fluorescent dye (FluoVolt), see also Supplementary Video 5. **A)** Optical traces obtained from the top (green) site shown in Fig. 6A,D), before (black) and after (colored) motion tracking and artifact compensation, c.f. left (green) trace in E). Residual illumination-related ,otion artifacts **B)** Optical traces obtained from the bottom (blue) site shown in Fig. 6A,D), before (black) and after (colored) motion tracking and artifact compensation, c.f. right (blue) trace in E).

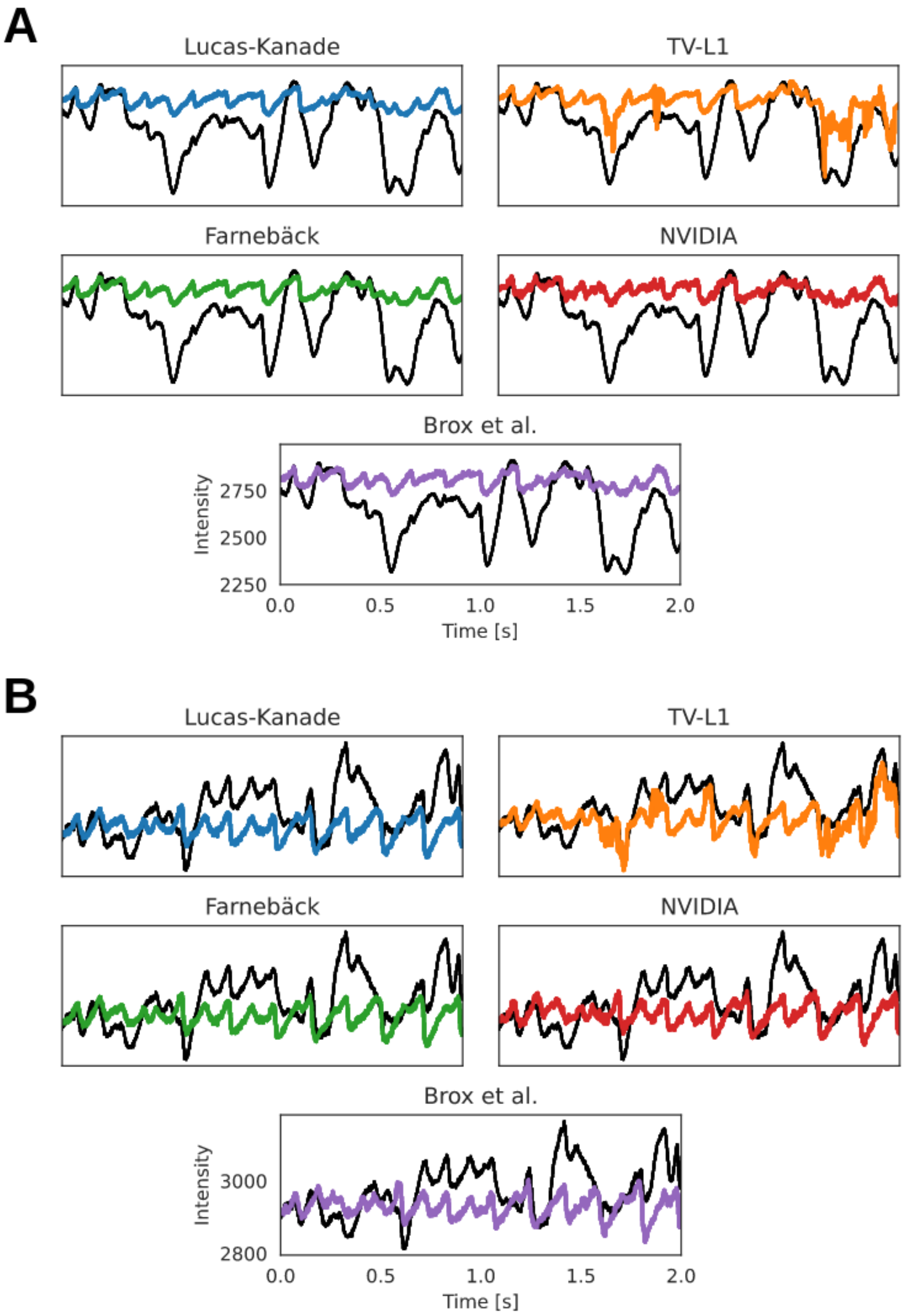

Figure S8. Comparison of motion-stabilized optical traces and optical maps obtained with 5 different GPU-based motion tracking algorithms imaging a beating rabbit heart during ventricular fibrillation with a Basler acA720-520um camera and voltage-sensitive fluorescent dye (Di-4-ANEPPS). **A)** Optical traces obtained from the top (green) site shown in Fig. 2A), before (black) and after (colored) motion tracking and artifact compensation, c.f. left (blue) trace in C). Residual illumination-related ,otion artifacts **B)** Optical traces obtained from the bottom (blue) site shown in Fig. 2A), before (black) and after (colored) motion tracking and artifact compensation, c.f. right (green) trace in C).
